# Addressing abortion through individual and community-level determinants: Evidence from Southern Ethiopia

**DOI:** 10.1371/journal.pone.0349603

**Published:** 2026-06-17

**Authors:** Amanuel Yoseph, Lakew Mussie, Mehretu Belayneh, Francisco Guillen-Grima

**Affiliations:** 1 School of Public Health, College of Medicine and Health Sciences, Hawassa University, Hawassa, Ethiopia; 2 Department of Epidemiology and Biostatistics, Schulich School of Medicine & Dentistry, Western University‌‌, London, Ontario, Canada; 3 Adare General Hospital, Hawassa City Administration, Hawassa‌‌, Ethiopia; 4 Department of Health Sciences, Public University of Navarra, Pamplona, Spain; Zurich University of Applied Sciences: ZHAW Zurcher Hochschule fur Angewandte Wissenschaften, SWITZERLAND

## Abstract

**Background:**

Abortion remains a significant public health concern in Ethiopia, contributing to maternal morbidity and mortality. Understanding the individual- and community-level determinants of abortion is essential to inform targeted interventions. This study aimed to assess the prevalence and multilevel determinants of abortion among women of reproductive age in Hawela Lida District, Southern Ethiopia.

**Methods:**

A community-based cross-sectional survey was conducted from February to March 2025 among 3,526 women of reproductive age who experienced a pregnancy within the 12 months preceding the survey. Data were collected using a structured, pretested questionnaire and analyzed with Stata v18. Multilevel mixed-effects logistic regression was employed to identify individual- and community-level determinants, adjusting for potential confounders. Adjusted odds ratios (AOR) with 95% confidence intervals (CI) were reported.

**Results:**

The overall prevalence of abortion was 18.5% (95% CI: 15.8–24.8). At the individual level, women without formal education were more likely to experience abortion than those with formal education (AOR = 2.61; 95% CI: 1.36–4.54). Women with unplanned pregnancies (AOR = 2.72; 95% CI: 1.62–4.68) and those who were non-autonomous in decision-making (AOR = 2.21; 95% CI: 1.51–5.02) also had higher odds of abortion. At the community level, rural residence (AOR = 2.19; 95% CI: 1.65–4.23), greater distance to the nearest health facility (AOR = 2.34; 95% CI: 1.21–5.01), and low mass media exposure (AOR = 1.82; 95% CI: 1.23–4.12) were significantly associated with abortion.

**Conclusion:**

Abortion in Hawela Lida District was associated with both individual and community factors, including women’s education, pregnancy planning, autonomy, place of residence, mass media exposure, and health facility accessibility. Integrated interventions that strengthen family planning, promote women’s empowerment, improve service accessibility, and provide accurate reproductive health information are urgently needed to reduce abortion prevalence and improve maternal health outcomes in Ethiopia.

## Introduction

Abortion remains a critical global public health concern, contributing significantly to maternal morbidity and mortality, particularly in low- and middle-income countries [1]. Although the global maternal mortality ratio has declined substantially over the past two decades, unsafe abortion continues to account for nearly 13% of maternal deaths worldwide [1,2]. Sub-Saharan Africa bears a disproportionate share of this burden, where restrictive abortion laws, limited access to safe services, and sociocultural barriers converge to increase women’s vulnerability [3]. Ethiopia, despite progressive policy reforms such as the 2005 revision of its abortion law, continues to face a high incidence of unsafe abortions, highlighting the persistence of systemic gaps in reproductive health service delivery [4,5].

Previous studies in Ethiopia and other parts of the world have identified a range of factors associated with abortion, spanning individual, household, and community levels [6,7]. At the individual level, education, economic status, and reproductive autonomy have consistently emerged as important determinants [8,9]. Unplanned pregnancies, often linked to limited contraceptive use and unmet family planning needs, remain a major driver of abortion globally [10]. At the community level, disparities between rural and urban settings, accessibility of health facilities, and exposure to mass media also shape reproductive decisions [11,12]. However, much of the existing evidence relies on single-level analyses, which fail to account for the hierarchical nature of determinants embedded within broader community structures [13]. This analytical limitation reduces the capacity to fully capture contextual influences on abortion practices.

In the Ethiopian context, research has largely concentrated on quantifying abortion prevalence and exploring proximate individual factors such as age, parity, and contraceptive use [14,15]. Far fewer studies have incorporated community-level determinants such as literacy, poverty, and geographic access to health services, despite their well-documented influence on maternal health outcomes [16]. Moreover, while the prevalence of abortion has been estimated in urban and facility-based settings, population-level evidence from rural districts remains scarce, leaving a significant gap in understanding the interplay between personal and structural determinants. This knowledge gap is particularly relevant in the Sidama Region, a predominantly rural area where maternal health outcomes continue to lag behind national averages.

Addressing abortion from both individual and community perspectives is crucial for designing effective interventions and policies. Understanding how community contexts -such as residence, access to infrastructure, and shared socio-economic conditions -interact with individual characteristics like education, autonomy, and pregnancy intention provides a more holistic view of abortion dynamics. Without such insights, policy responses risk remaining fragmented, addressing isolated determinants without acknowledging their broader socio-ecological underpinnings [17,18]. By employing multilevel modeling, researchers can disentangle these cross-cutting influences and provide stronger evidence to guide programming and policy at multiple levels.

The present study seeks to address these gaps by investigating the individual- and community-level determinants of abortion among women of reproductive age in the Northern Zone of Sidama Region, Southern Ethiopia. Specifically, it applies a multilevel mixed-effects logistic regression framework to disentangle the relative contributions of personal, household, and contextual factors to abortion outcomes. By generating robust evidence from a large, community-based sample, this study aims to inform integrated maternal health strategies that extend beyond individual-level risk factors to address the broader structural and societal conditions shaping women’s reproductive health.

## Methods

### Study setting‌‌

This study was conducted in Hawela Lida District, one of 30 districts in Ethiopia’s Sidama Region, located approximately 289 km south of Addis Ababa. The district comprises 11 rural and two urban *kebeles* (the smallest administrative units). In 2024, the estimated population was 131,848 across 24,281 households, with women of reproductive age (15–49 years) representing 24.3% of the population. Agriculture constitutes the primary livelihood, with major crops including enset (false banana), maize, coffee, khat, barley, haricot beans, sweet potatoes, and indigenous cabbage. Health infrastructure comprises 20 government health posts, four health centres, five private medium clinics, two NGO-run facilities, and six private pharmacies, staffed by 482 health professionals. Health posts, managed by Health Extension Workers (HEWs), provide essential maternal and reproductive health services, including health education, family planning, antenatal and postnatal care. Overall maternal health service coverage in the district is estimated at 70%.

### Study design and period

A community-based cross-sectional survey was conducted between February 1 and March 30, 2025. The study adhered to the Strengthening the Reporting of Observational Studies in Epidemiology (STROBE) guidelines (S1 File).

### Source and study population

The source population comprised all women of reproductive age residing in the district. The study population included women aged 15–49 years who experienced a pregnancy within the 12 months preceding the survey and had lived in the district for at least twelve months.

### Eligibility criteria

Women were eligible if they had resided in Hawela Lida District for at least one year and reported a pregnancy that ended in abortion, stillbirth and live birth within the same period. This criterion ensured that participants had sufficient exposure to the local community context, allowing for an accurate assessment of both individual- and community-level determinants. Women who were critically ill, mentally incapacitated, or unable to provide informed consent were excluded.

### Sample size determination

We computed the sample size using OpenEpi version 3.0. The initial sample size was estimated using a single population proportion formula, assuming a 15% prevalence of abortion from previous study [19], a 95% confidence level, and a 5% margin of error. This yielded a base sample size of 392. To account for the multistage cluster sampling design, we applied a design effect (DEFF) of 2.0. The design effect was considered appropriate to adjust for intra-cluster correlation within kebeles and to improve the representativeness of estimates under a clustered sampling framework. We also added a 10% non-response rate, which increased the minimum required sample size to 863.

In addition, because the study aimed to examine both individual- and community-level determinants using multilevel mixed-effects logistic regression, we further increased the sample size to ensure sufficient statistical power to detect contextual (cluster-level) effects. This step was necessary to improve precision in estimating both fixed and random effects in the hierarchical data structure. Finally, after adjusting for clustering, non-response, and multilevel analytical requirements, the final sample size was increased to 3,540 women, which was considered adequate to address both individual- and community-level determinants in the study.

### Sampling procedure

**Fig 1:**
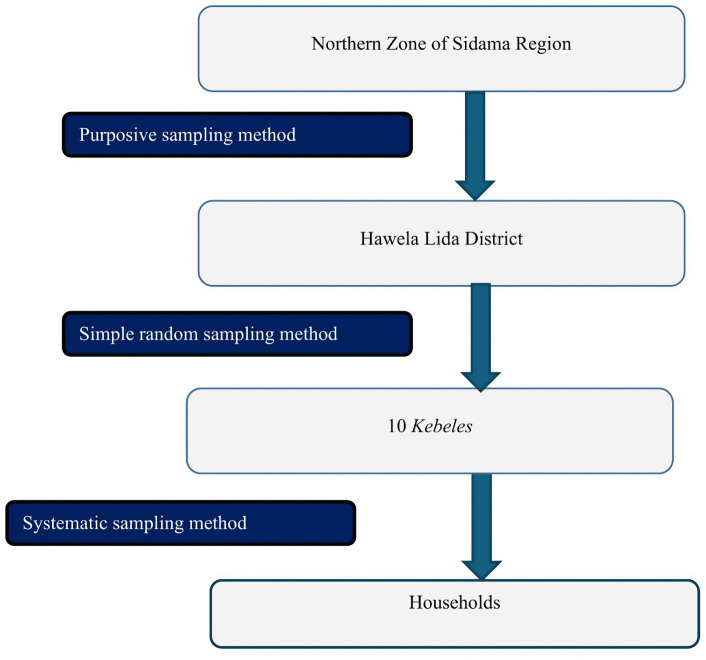
Flowchart of the multistage sampling procedure for the study of abortion determinants among women of reproductive age in Hawela Lida District, Sidama Region, Ethiopia, 2025.

A multistage sampling strategy was applied. First, Hawela Lida District was purposively selected due to its diverse rural-urban population, ease of supervising data collection, and administrative priority because of a reported high prevalence of abortion. In the second stage, 10 out of the district’s 13 *kebeles* were randomly selected. Within these *kebeles*, a household census identified all women who had delivered in the previous 12 months, forming the sampling frame. In the third stage, eligible households were systematically selected proportional to *kebele* size. If multiple eligible women were present in a household, one was randomly chosen. Households were classified as non-respondents after three unsuccessful contact attempts (Fig 1).

### Study variables

#### Outcome variable.

The primary outcome of interest was abortion within the past 12 months, defined as a pregnancy that ended in termination – either induced or spontaneous – as reported by respondents. Women were classified as having experienced an abortion if they answered “yes” to the relevant question; otherwise, they were coded as “no.”

#### Individual-level predictors.

Several sociodemographic, reproductive, and behavioral factors were examined. **Educational attainment** was measured as the highest level of formal schooling completed and categorized as no education, primary, secondary, or higher education. **Age** was recorded in completed years and analyzed as both a continuous variable and grouped into conventional reproductive-age categories (15–19, 20–24, 25–29, 30–34, and 35–49 years). **Marital status** was classified as married/cohabiting versus not married (single, divorced, or widowed). **Parity** was defined as the total number of pregnancies reaching a viable gestational age (≥28 weeks), including both live births and stillbirths. This variable was treated as continuous for analysis and categorized for descriptive purposes (0, 1–2, ≥ 3). **Wealth quintiles** were derived using principal component analysis (PCA) of household assets. Variables with very low (<5%) or very high (>95%) prevalence, inadequate sampling adequacy (Kaiser-Meyer-Olkin <0.5), low communalities (<0.5), or complex loadings (>0.4 on multiple components) were excluded. The resulting factor scores were ranked into five quintiles: lowest, second, middle, fourth, and highest. **Obstetric danger signs** during pregnancy were measured using nine self-reported complications, including severe bleeding, convulsions, prolonged labor, high fever, and blurred vision. Each of the nine self-reported obstetric danger signs during pregnancy was coded as “1 = Yes” if experienced and “0 = No” if not. Responses of “I don’t know” was treated as “0 = No” for consistency.

#### Community-level predictors.

Respondents were nested within *kebeles*, the smallest administrative unit in Ethiopia, to account for contextual influences. Community-level variables were constructed by aggregating individual responses within each *kebele*. These included the proportion of women with formal education (community education) and the proportion of households below the poverty threshold (community poverty). Each *kebele* was assigned a unique identifier and modeled as a random intercept to capture unobserved heterogeneity. **Community-level mass media** use was calculated as the proportion of women in each *kebele* who reported regularly listening to the radio, watching television, or reading newspapers. *kebeles* where more than 50% of participants used at least one form of mass media were classified as “high,” and those with 50% or fewer as “low.”

#### Operationalization and coding.

All binary variables were coded as 1 (“yes”) and 0 (“no”). For community-level variables, aggregate proportions were calculated as continuous measures. Detailed variable definitions are provided in S2 File, Table 1.

**Table 1:** Socio-demographic and economic characteristics of mothers in Hawela Lida district, Southern Ethiopia, 2025 (N = 3,526).

Variable	Category	n	%
**Maternal age, y**	15–49 (mean ± SD)	26.0 ± 4.6	—
**Ethnicity**	Sidama	3,328	94.3
	Amhara	125	3.5
	Wolayita	38	1.1
	Gurage	35	1.0
**Religion**	Protestant Christian	2,990	84.8
	Orthodox Christian	236	6.7
	Catholic	172	4.9
	Muslim	99	2.8
	Other	29	0.8
**Maternal education**	Cannot read/write	375	10.6
	Read/write only	75	2.1
	Primary (1–8)	2,486	70.5
	Secondary (9–12)	364	10.3
	College diploma	119	3.4
	University ≥ degree	107	3.0
**Maternal occupation**	Housewife	3,041	86.2
	Government employee	278	7.9
	Merchant	207	5.9
**Marital status**	Married	3,499	99.2
	Div./sep./widowed	27	0.8
**Husband occupation**	Farmer	1,935	54.9
	Merchant	1,029	29.2
	Government employee	311	8.8
	Daily labourer	172	4.9
	Private employee	21	0.6
	Other	58	1.6
**Husband education**	Cannot read/write	120	3.4
	Read/write only	473	13.4
	Primary (1–8)	1,932	54.8
	Secondary (9–12)	707	20.0
	College diploma	159	4.5
	University ≥ degree	135	3.8
**Household size**	1–5 members	2,777	78.8
	> 5 members	749	21.2
**Mass-media exposure**	Yes	1,399	39.7
	No	2,127	60.3
**Wealth quintile**	Lowest	717	20.3
	Second	730	20.7
	Middle	682	19.3
	Fourth	669	19.0
	Highest	728	20.7

### Data collection procedures

Data were collected using a structured, interviewer-administered questionnaire adapted from previous reproductive health and abortion studies (S3 File). The tool was developed in English, translated into *Sidaamu Afoo* (Local language), and back-translated to ensure consistency. Data collectors and supervisors, all fluent in *Sidaamu Afoo* and trained in ethical and interview procedures, received two days of training. Pretesting on 5% of the sample in a neighboring district allowed refinement of the instrument. Data were collected face-to-face at participants’ homes using the Open Data Kit mobile application (S4 File). Daily checks ensured completeness and accuracy before secure archiving on the Kobo Toolbox server.

### Statistical analysis

Descriptive analyses summarized categorical variables as frequencies and percentages and continuous variables as means ± standard deviation. Wealth index was derived using PCA; detailed procedures are provided in S2 File. Multi-level mixed-effects logistic regression models were fitted to identify individual- and community-level determinants of abortion, accounting for clustering at the *kebele* level. Four models were constructed: Model 0 (empty), Model 1 (individual-level determinants), Model 2 (community-level determinants), and Model 3 (combined determinants). Model selection was guided by Akaike’s Information Criterion (AIC), Bayesian Information Criterion (BIC), log-likelihood, intraclass correlation coefficients (ICC), and median odds ratios (MOR). Variables with p < 0.10 in bivariable analysis or those with established clinical and social relevance were included in the multivariable model. Interaction terms were used to assess effect modification, and multicollinearity was checked using variance inflation factors (VIF < 5). Associations were reported as adjusted odds ratios (AORs) with 95% confidence intervals, with statistical significance defined as 95% CI not crossing 1.

### Ethical considerations

Ethical approval was obtained from the Institutional Review Board of the College of Medicine and Health Sciences, Hawassa University with Reference number: IRB/027/17. Additional permissions were obtained from the Sidama Regional Health Bureau, Hawela Lida District Health Office, and *kebele* administrations. Written informed consent was obtained from all participants after providing detailed information about the study’s objectives, procedures, potential risks, and benefits. No personal identifiers were collected, and data were stored on password-protected servers accessible only to the research team, ensuring confidentiality.

## Results

### Study population

A total of 3,526 mothers participated in the study (Table 1). The mean maternal age was 26.0 ± 4.6 years, ranging from 15 to 49 years. The majority were of Sidama ethnicity (94%) and identified as Protestant Christians (85%), with nearly all currently married (99%). While 70% of participants had completed at least primary education, only 16.7% attained secondary education or higher. Most women were housewives (86%), whereas the predominant occupation among husbands was farming (55%). Household size was typically small, with four in five families comprising five or fewer members. Approximately 40% of respondents reported regular exposure to mass media. The asset-based wealth index was relatively evenly distributed across quintiles.

### Reproductive and obstetric characteristics

The mean age at first marriage and first pregnancy were 21.2 ± 3.1 and 22.4 ± 3.2 years, respectively (Table 2). Most women (69%) had experienced two to four pregnancies, and 18.5% reported at least one prior abortion. Previous stillbirth was rare (1.6%). Although 83.4% of pregnancies were planned, 9.5% of mothers reported experiencing at least one obstetric danger sign during pregnancy.

**Table 2:** Reproductive and obstetric characteristics of mothers in Hawela Lida District, Southern Ethiopia, 2025 (N = 3,526).

Variable	Category (or mean ± SD)	n	%
**Age at first marriage, y**	21.2 ± 3.1	—	—
**Age at first pregnancy, y**	22.4 ± 3.2	—	—
**Number of gravidities**	1	911	25.8
	2–4	2,439	69.2
	≥ 5	176	5.0
**Previous abortion**	Yes	652	18.5
	No	2,874	81.5
**Previous stillbirth**	Yes	55	1.6
	No	3,471	98.4
**Pregnancy planned**	Yes	2,942	83.4
	No	584	16.6
**Obstetric danger sign during pregnancy**	Yes	334	9.5
	No	3,192	90.5

### Prevalence of abortion

**Fig 2:**
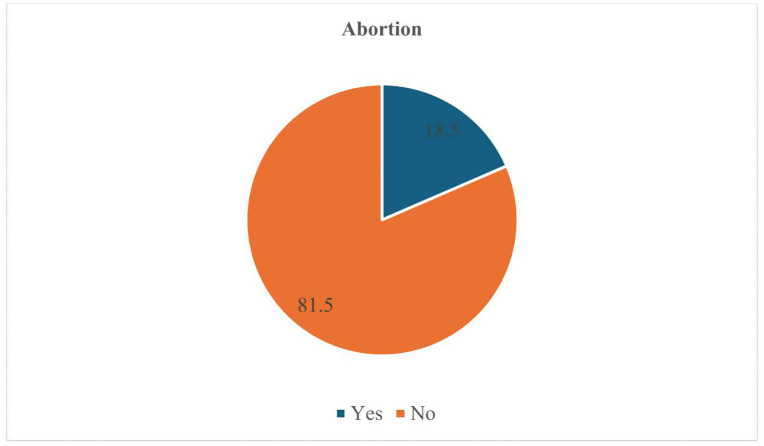
Prevalence of abortion among women of reproductive age (15–49 years) in Hawela Lida District, Southern Ethiopia, 2025.

Among the 3,526 women surveyed, the overall prevalence of abortion was 18.5% (95% CI: 15.8–24.8) (Fig 2). This indicates that nearly one in five women of reproductive age in Hawela Lida District experienced an abortion within the preceding 12 months.

The bivariable analysis (Table 3) was used to screen candidate variables for inclusion in the multilevel models presented in Table 4.

**Table 3:** Bivariate logistic regression analysis of determinants of abortion among women, Hawela Lida District, 2025 (n = 3,526).

Variables	Category	Abortion Yes n (%)	Abortion No n (%)	P-value
**Individual-level determinants**				
Women’s education status	No formal	260 (33.8)	509 (66.2)	<0.001**
	Formal	392 (14.3)	2,343 (85.7)	
Pregnancy status	Unplanned	305 (35.3)	558 (64.7)	<0.001**
	Planned	347 (12.3)	2,316 (87.7)	
Woman’s decision-making power	Non-autonomous	572 (27.9)	1,476 (75.0)	0.002 *
	Autonomous	80 (10.4)	690 (89.6)	
Husband’s education	No formal	50 (41.7)	70 (58.3)	0.03 *
	Formal	602 (17.4)	2,804 (82.6)	
Women’s occupation	Housewife	512 (16.8)	2,528 (83.2)	0.06
	Government employee	70 (25.2)	208 (74.8)	
	Merchant	20 (74.1)	7 (25.9)	
Exposure to mass media	Yes	200 (14.3)	1,199 (85.7)	0.09
	No	452 (21.2)	1,675 (78.8)	
Household wealth quintile	Lowest	150 (20.9)	567 (79.1)	0.07
	Second	145 (19.9)	585 (80.1)	
	Middle	125 (18.3)	557 (81.7)	
	Fourth	110 (16.4)	559 (83.6)	
	Highest	122 (16.8)	606 (83.2)	
Age at first pregnancy	–	–	–	0.04*
Road access	Yes	400 (18.0)	1,820 (82.0)	0.06
	No	252 (19.2)	1,054 (80.8)	
**Community-level determinants**				
Place of residence	Urban	104 (16.0)	546 (84.0)	<0.001**
	Rural	520 (28.8)	1,285 (71.2)	
Community-level distance to nearest health facility	Big problem	253 (33.6)	499 (66.4)	<0.001**
	Not big problem	399 (14.4)	2,375 (85.6)	
Community-level mass media use	High	415 (15.6)	2,242 (84.4)	<0.001**
	Low	237 (27.3)	632 (72.7)	
Community-level poverty	High	300 (18.1)	1,355 (81.9)	0.04*
	Low	352 (18.9)	1,511 (81.1)	
Community-level female literacy	High	250 (17.5)	1,177 (82.5)	0.02*
	Low	402 (19.5)	1,689 (80.5)	

*: significant association (*p* < 0.05); **: highly significant association (*p* < 0.01); ©: continuous variable. P-values were calculated using the chi-square test for categorical variables and the t-test for numerical variables.

**Table 4:** Multilevel logistic regression analysis of determinants of abortion among women, Hawela Lida District, 2025 (N = 3,526).

Variables	Category	Model 1 (individual AOR)	Model 2 (Community AOR)	Model 3 (Final AOR)	P-value
**Individual-level determinants**					
Women’s education status	No formal	3.05 (1.44-8.62)	–	2.61 (1.36-4.54)	<0.001**
	Formal	Ref	–	Ref	
Pregnancy status	Unplanned	3.65 (1.99-7.68)	–	2.72 (1.62-4.68)	<0.001**
	Planned	Ref	–	Ref	
Woman’s decision-making power	Non-autonomous	2.99 (1.38-5.90)	–	2.21 (1.51-5.02)	0.02*
	Autonomous	Ref	–	Ref	
Husband’s education	No formal	1.06 (0.72-1.47)	–	0.87 (0.62-1.32)	0.25
	Formal	Ref	–	Ref	
Women’s occupation	Housewife	Ref	–	Ref	
	Government employee	1.04 (0.66-1.43)	–	1.02 (0.64-1.44)	0.32
	Merchant	0.87 (0.59-1.61)	–	0.82 (0.56-1.88)	0.41
Exposure to mass media	Yes	1.06 (0.68-1.47)	–	0.95 (0.66-1.38)	0.21
	No	Ref	–	Ref	
Household wealth quintile	Lowest	Ref	–	Ref	
	Second	0.91 (0.61-1.28)	–	0.92 (0.54-1.30)	0.09
	Middle	0.86 (0.54-1.27)	–	0.92 (0.68-1.26)	0.08
	Fourth	0.83 (0.68-1.21)	–	0.55 (0.41-1.19)	0.04
	Highest	0.87 (0.69-1.16)	–	0.95 (0.67-1.21)	0.07
Age at first pregnancy	–	1.05 (0.97-1.06)	–	0.98 (0.95-1.06)	
Road access	Yes	1.09 (0.65-1.49)	–	1.02 (0.66-1.52)	0.15
	No	Ref		Ref	
**Community-level determinants**					
Place of residence	Urban	–	Ref	Ref	
	Rural	–	2.49 (1.18-5.72)	2.19 (1.65–4.23)	0.03*
Community-level distance to nearest health facility	Big problem	–	3.02 (1.42-6.42)	2.34 (1.21-5.01)	<0.001**
	Not big problem	–	Ref	Ref	
Community-level mass media use	High	–	Ref	Ref	
	Low	–	2.08 (1.58-5.83)	1.82 (1.23-4.12)	0.03*
Community-level poverty	High	–	Ref	Ref	
	Low	–	1.07 (0.68-1.53)	1.04 (0.68-1.44)	0.18
Community-level female literacy	High	–	Ref	Ref	
	Low	–	1.08 (0.81-1.47)	1.05 (0.69-1.46)	0.16

*: significant association (*p* < 0.05); **: highly significant association (*p* < 0.01); AOR: adjusted odds ratio; CI: confidence interval; ©: continuous variable; Ref: reference group.

### Determinants of abortion among women of reproductive age

The multivariable analysis identified several individual- and community-level factors that were significantly associated with abortion among women of reproductive age in Hawela Lida District (Table 4).

At the individual level, women without formal education were more than twice as likely to experience abortion compared to those with formal education (AOR = 2.61; 95% CI: 1.36–4.54, p < 0.01). Similarly, women with unplanned pregnancies had higher odds of abortion than those with planned pregnancies (AOR = 2.72; 95% CI: 1.62–4.68, p < 0.01). Women who were non-autonomous in decision-making also faced increased odds of abortion compared to autonomous women (AOR = 2.21; 95% CI: 1.51–5.02, p < 0.05).

At the community level, women living in rural areas were more likely to experience abortion compared to those in urban areas (AOR = 2.19; 95% CI: 1.65–4.23, p < 0.05). Communities reporting greater distance-related barriers to the nearest health facility (“big problem”) were also associated with higher odds of abortion (AOR = 2.34; 95% CI: 1.21–5.01, p < 0.01). Furthermore, communities with low mass media exposure had increased odds of abortion compared to those with higher exposure (AOR = 1.82; 95% CI: 1.23–4.12, p < 0.05).

### Multilevel model performance and between-*kebele* variability in abortion determinants

The multilevel logistic regression analysis demonstrated a significantly better fit to the data compared with the conventional logistic regression model (p < 0.001). The intraclass correlation coefficient revealed that 15.71% of the variance in abortion was explained by clustering at the *kebele* level. Notably, even after adjusting for both individual- and community-level characteristics, *kebele*-level variations remained statistically significant, contributing to 17.50% of the residual variability. The Median Odds Ratio (MOR) was estimated at 2.21, highlighting substantial heterogeneity between *kebeles*. Furthermore, the influence of women’s education on abortion outcomes varied significantly across *kebeles* (variance = 0.07; 95% CI: 0.03–1.32), highlighting important contextual differences.

Model fit statistics further supported the robustness of the final specification. Comparative assessment using the AIC, BIC, and log-likelihood values confirmed the superiority of the final multilevel model over simpler alternatives. Specifically, the empty model yielded AIC = 1042.32, BIC = 1066.23, and log-likelihood = –520.72, whereas the final model achieved AIC = 1001.50, BIC = 1014.66, and log-likelihood = –440.63. Collectively, these indices affirm the enhanced explanatory power and reliability of the multilevel framework in capturing the determinants of abortion across *kebeles*.

## Discussion

### Prevalence of abortion

The prevalence of abortion in the current study was 18.5% (95% CI: 15.8–24.8), indicating that nearly one in five women of reproductive age in Hawela Lida District experienced an abortion within the preceding 12 months. This estimate is notably higher than the national prevalence reported in the 2016 Ethiopian Demographic and Health Survey (EDHS), which documented approximately 10% of women having terminated a pregnancy within five years [19]. The observed discrepancy may reflect methodological differences, as the present study employed a community-based survey design with a 12-month recall period, minimizing recall bias and providing a more precise estimate.

Comparisons within Ethiopia indicate variability in abortion prevalence. For instance, studies conducted in Addis Ababa reported prevalence of 19.6% [20], closely mirroring our findings, whereas rural regions such as Amhara and Oromia exhibited lower prevalence, ranging from 12% to 15% [21,22]. Globally, the observed prevalence aligns with sub-Saharan African estimates, where abortion prevalence range from 14% to 22% [23,24]. Conversely, countries with more liberal abortion legislation and higher contraceptive coverage, such as South Africa and Ghana, report comparatively lower abortion prevalence, highlighting the role of accessible reproductive health services in preventing unintended pregnancies [25]. These patterns highlight that despite improvements in maternal health services, unmet contraceptive needs and gaps in women’s reproductive autonomy continue to contribute to high abortion rates in Ethiopia.

### Individual-level determinants

Women’s education was significantly associated with abortion. Women without formal education exhibited more than twice the odds of abortion compared to their educated counterparts. This finding concurs with studies in Ethiopia [21,26] and Nigeria [27], where higher educational attainment enhances reproductive health literacy, contraceptive use, and informed decision-making. Conversely, in contexts such as Uganda [28] and Kenya [29], educated women may be more empowered to seek abortion in response to unplanned pregnancies, demonstrating that the education–abortion relationship is context-dependent, mediated by cultural norms, legal frameworks, and health system accessibility.

Pregnancy intention also emerged as a significant associated factor with abortion‌‌. Women who reported unplanned pregnancies had lower odds of abortion compared with those whose pregnancies were planned. This finding suggests that, in this setting, pregnancies that were initially unintended were not necessarily followed by termination and may instead have been continued due to social, cultural, or personal considerations. Similar patterns have been documented in previous studies conducted in Ethiopia [30–32] and rural Tanzania [32]. These findings highlight the complex relationship between pregnancy intention and abortion and underscore the importance of strengthening access to family planning information and modern contraceptive methods to reduce unintended pregnancies and support informed reproductive decision-making [23,30].

Women’s autonomy significantly associated with abortion outcomes. Non-autonomous women were more than two times more likely to experience abortion compared to autonomous women. While autonomy generally empowers women to prevent unintended pregnancies, in contexts where abortion services are accessible, autonomy may facilitate safe abortion when necessary. Similar patterns have been observed in Addis Ababa [20] and Ghana [33], suggesting that autonomy operates as both an increasing and decreasing factor depending on sociocultural norms and healthcare availability.

### Community-level determinants

Community factors also associated with abortion outcomes. Rural residence was associated with higher odds of abortion, consistent with studies from Ethiopia [21,31,34] and Kenya [35], where rural women face geographic, infrastructural, and sociocultural barriers limiting access to reproductive health services [36,37]. Proximity to healthcare facilities was inversely associated with abortion; women perceiving minimal distance barriers had lower odds of abortion (AOR = 0.71; 95% CI: 0.47–0.89), corroborating studies from Ethiopia and Malawi [22,38]. Accessible facilities facilitate timely contraceptive counseling and antenatal care, reducing unintended pregnancies and unsafe abortions.

Lower community-level mass media exposure was associated with higher odds of abortion. Although mass media is generally expected to promote awareness of family planning and reproductive health services, limited exposure in this context may reflect reduced access to accurate reproductive health information, including knowledge about contraception and safe pregnancy prevention methods. As a result, women living in communities with low mass media exposure may face a higher risk of unintended pregnancies, which can subsequently increase the likelihood of abortion. Comparable findings have been reported in Nigeria [27] and Uganda [28], suggesting that mass media plays an important role in shaping reproductive health knowledge and decision-making among women.

It is important to recognize that abortions result from multiple causes, including biological factors such as chromosomal abnormalities, which primarily contribute to early spontaneous losses and are largely unaffected by social or community-level influences. However, in community-based populations like Hawela Lida District, many abortions are associated with preventable or socially mediated factors, including unintended pregnancies, limited reproductive autonomy, restricted access to healthcare, and sociocultural pressures. Our multilevel analysis highlights that community-level variables such as rural residence, proximity to health facilities, and exposure to mass media significantly associated with abortion outcomes, reflecting the role of social and environmental determinants in reproductive behavior. While biological factors remain important, these findings emphasize that a substantial portion of abortions in this setting can be mitigated through interventions targeting social, behavioral, and healthcare-related determinants. This distinction highlights the value of assessing both individual- and community-level factors when designing policies and programs to reduce abortion prevalence.

### Methodological strengths and limitations

Key methodological strengths include the large sample size (N = 3,526), which provided sufficient statistical power to assess individual- and community-level determinants. The use of multilevel mixed-effects modeling enabled robust estimation by accounting for clustering effects and disentangling contextual influences from individual-level determinants [39,40]. Moreover, the 12-month recall period minimized recall bias, enhancing reliability.

However, limitations include potential underreporting due to stigma and social desirability bias, which could have led to conservative prevalence estimates. The cross-sectional design precludes causal inference, and community-level variables derived from aggregated individual responses may not fully capture broader structural determinants, such as facility capacity or legal enforcement. Qualitative insights into sociocultural factors influencing abortion decisions were also absent.

### Policy and practice implications

The findings emphasize the need for comprehensive, multi-level interventions. Strengthening family planning services, particularly in rural areas and among less educated and non-autonomous women, is paramount. Infrastructure development, including improved road networks and expanded health facilities, can reduce geographic barriers. Mass media campaigns should be carefully tailored to promote contraceptive uptake while ensuring responsible reproductive decision-making. Policy efforts should continue to expand safe abortion services within legal frameworks, integrate post-abortion care into broader reproductive health programs, and promote women’s empowerment.

## Conclusion

Abortion in Hawela Lida District was associated with both individual- and community-level determinants. Low education, unplanned pregnancy, limited autonomy, rural residence, restricted healthcare access, and low mass media exposure significantly increased abortion prevalence. Addressing these determinants through integrated, multilevel interventions is critical for reducing abortion prevalence and improving maternal health outcomes. Policies that enhance family planning, expand safe abortion services, and empower women are urgently needed in Ethiopia and similar settings.

## Supporting information

S1 File STROBE reporting guidelines for cluster RCTs checklist. (DOCX)

S2 File Detail information from methods and results sections. (DOCX)

S3 File English version study questionnaire. (DOCX)

S4 File De-identified SPSS dataset, which was authorized to be available by the institutional ethics committee and supported by informed consent from all study participants (Sav). (XLSX)
