# Supplementary material for: Addressing abortion through individual and community-level determinants: Evidence from Southern Ethiopia
Source: PLoS One. 2026 Jun 17;21(6):e0349603. doi: 10.1371/journal.pone.0349603 (PMC13274891; doi:10.1371/journal.pone.0349603)
Supplement: S3 File — (DOCX) [file pone.0349603.s003.docx]

**ANNEXES**

**Annex I: Information sheet**

**Hawassa University**

**College of Medicine and Health Sciences**

**School of Public Health**

**Project title: Addressing Abortion through Individual and Community-Level Determinants: Evidence from Southern Ethiopia**

Hello! My name is _------------------------------------------------------_. I am a data collector in a study conducted about maternal health service utilization under the College of Medicine and Health Sciences of Hawassa University. We are conducting a study to improve maternal health service utilization in your community.

**Goal of the study**

The objective of this study is to improve maternal health service utilization. The study will be conducted for six months. After this baseline survey, selected mothers in some villages will be provided with health education regarding maternal health topics.

At the end of intervention, we will test or check how well the education has assisted to improve MHSU in your villages by comparing to other villages that did not obtain education. The data obtained in this study will be valuable to prepare the education regarding maternal health topics.

**Significance of the study and method of participant selection**

The aim of this study is to generate information about the maternal health service utilization among women of reproductive age in North Zone of Sidama region. The study may help public health planners, stakeholders, policy makers, responsible bodies, and others to take actions based on the finding. The study comprises various intimate and private questions. We randomly selected you to help us by participating in this study as the data you offer us will be helpful in averting maternal and child death and illness. Interview will not take more than 40 minutes.

**Risks and discomfort**

We assure you that there is no risk or harm in participating in this study. The study comprises various friendly and private questions. However, you may refuse to respond to any of the queries if you feel uncomfortable.

**Benefits**

Your involvement will assist us to find out more regarding maternal health issue in the villages and to provide education to women in your communities. If this study revealed that education is useful in increasing maternal health by improving proper use of MHS, the government and other stakeholders will be able to improve maternal health in other communities in this setting and other part of the country.

**Incentives**

There is no incentive or payment for participating in this research. Nonetheless, we thank you very much for your involvement.

**Confidentiality**

All information will be kept confidentially. Name of the participant will not be written or specified. Your privacy will also be protected, and no one shall know your response.

**Right to withdraw or refuse**

You have full right whether to participate in this study. You may respond to all questions, or you may not answer the questions you don’t want to, or you may quit your participation totally at any time you want. You can ask any questions which are not clear to you. Moreover, we assure you that refusing to participate will not affect your future treatment at the health institution or elsewhere in any way.

**Who to contact**

You can ask any questions which are not clear to you now or later. If you wish to ask queries later, you may contact: Amanuel Yoseph, Hawassa University, Telephone: 0915683578.

**Annex II: Informed Consent**

I have been requested to take part in the research and read the foregoing information, or it has been read to me in the language I comprehend and understand all conditions stated above. I have had the opportunity to ask questions about it and any questions I have been asked to have been answered to my satisfaction. I consent voluntarily to be a participant in this study and understand that I have the right to withdraw from the interview at any time without in any way affecting my right.

Are you willing to participate in this study?

Yes □ No □

If “Yes”, study participant’s ID……………………Signature……………. Date………….

And proceed with the interview.

If “No” ...........thank you and end.

Name of the principal investigator: Amanuel Yoseph

Cell phone Number - +251-915683578 E mail: [amanuelyoseph45@gmail.com](mailto:amanuelyoseph45@gmail.com)

Name of interviewer___________________ Signature__________

Data of interviewer (Ethiopia calendar) _____/_____/_____

Result of interview: 1. Complete 2. Refused 3. Partially complete 4. Respondent not available

Cheeked by supervisor: Name______________________ Data_____/___/____

**Annex III: English version questionnaire**

**Hawassa University**

**College of Medicine and Health Sciences**

**School of Public Health**

***Questionnaire designed to study the Addressing Abortion through Individual and Community-Level Determinants: Evidence from Southern Ethiopia***

**PART I: General Information**

01. Name of Woreda: _________

02. Name of Kebele: ________________

03. Name of Gote __________________

04. House number ____________________

05. Individual Id __________________

06. Date of interview: ____________time interview started __________ ended at ___________

07. Respondent available on:

1. 1^st^ visit.

2. 2^nd^ visit.

3. 3^rd^ visit.

08: Data collection phase 1. Baseline (before intervention) 2. Endline (post-intervention)

09: Post-intervention data collection 1. Study participants from intervention group

2. Study participants from control group

**Module I: Socio-demographic characteristics of study participants**

**Instruction:** First, tell interviewee that you are going to ask her questions about herself. Second, properly explain to the study participant each question to elicit genuine response from the respondents. Then, request her to correctly respond to the questions. Please write the responses based on the respondents genuine responses (in field to be filled by data collectors).

| S.no | Questions | Response categories | Skip to | Remark |
| --- | --- | --- | --- | --- |
| 101 | What is your age (in completed years)? | __________ years |  |  |
| 102 | To which ethnic group do you belong? | \| 1. Sidama  2. Amhara  3. Oromo  4. Gurage  5. Wolayita  88.Other,specify_____ \| \| --- \| |  |  |
| 103 | What is your religious affiliation?  (encircle the response) | 1. Protestant Christian  2. Orthodox Christian  3. Catholic  4. Muslim  5.Other (specify) __________ |  |  |
| 104 | What is your highest educational level you have completed? | 1. Cannot read and write  2. Can read and write only  3. Primary education(1-8)  4. Secondary education(9-12)  5. College diploma  6. College/university degree and above |  |  |
| 105 | What is your occupation? | 1. Housewife  2. Farmer  3. Governmental employee  4. Merchant  5. Housemaid  6. Other (specify)……………… |  |  |
| 106 | What is your marital status? | 1. Not ever married…………….  2. Married  3. Divorced  4. Separated  5. Widowed  6. Cohabiting | Q110 |  |
| 107 | What is your husband‘s age in complete years? | _______________ |  |  |
| 108 | What is your husband‘s occupation? | 1. Governmental employee  2. Merchant  3. Farmer  4. Daily labourer  5. NGO employee  6. Private organization employee  7. Other (specify)……………… |  |  |
| 109 | What is your husband‘s educational status? | 1. Cannot read and write  2. Can read and write only  3. Primary education(1-8)  4. Secondary education(9-12)  5. College diploma  6. College/university degree and above |  |  |
| 110 | What is your family size? (In number) | ________ |  |  |
| 111 | Which social media do you use? | 1. Listens the radio 2. Watches the television 3. Reads the newspaper 4. All 5. Others |  |  |

**Module II: Household wealth index of study participants**

| S.no | Questions | Response categories | Skip to | Remark |
| --- | --- | --- | --- | --- |
| 201 | What is the main source of drinking water for members of your household? | 1. Piped water 2. Tube well or Borehole 3. Protected well 4. Unprotected well 5. Protected spring 6. Unprotected spring 7. Rain water 8. Surface water (River/dam) 9. Lake/pond/stream/canal 10. Bottled water |  |  |
| 202 | What is the main source of water used by your household for other purposes such as cooking and handwashing? | 1. Piped water 2. Tube well or Borehole 3. Protected well 4. Unprotected well 5. Protected spring 6. Unprotected spring 7. Rain water 8. Surface water (River/dam) 9. Lake/pond/stream/canal 10. Bottled water |  |  |
| 203 | Where is that water source located? | 1. In own dwelling 2. In own yard/plot 3. Elsewhere |  |  |
| 204 | How long does it take to go there, get water, and come back? | Minutes . . . . . . . . . . . . . . . .  998. Don’t know |  |  |
| 205 | In the past two weeks, was the water from this source not available for at least one full day? | 1. Yes 2. No   998. Don’t know |  |  |
| 206 | Do you do anything to the water to make it safer to drink? | 1. Yes 2. No   998. Don’t know |  |  |
| 207 | What kind of toilet facility do members of your household usually use?  IF NOT POSSIBLE TO DETERMINE, ASK PERMISSION TO OBSERVE THE FACILITY. | 1. Flush or pour flush toilet 2. Ventileted improved pit latrine 3. Pit latrine with slab 4. Pit latrine without slab/open pit 5. Compositing toilet 6. Bucket toilet 7. Hanging toilet 8. No facility/bush/field |  |  |
| 208 | Do you share this toilet facility with other households? | 1. Yes 2. No |  |  |
| 209 | Including your own household, how many households use this toilet facility? | 998. Don’t know |  |  |
| 210 | Where is this toilet facility located? | 1. In own dwelling  2. In own yard/plot  3. Elsewhere |  |  |
| 211 | What type of fuel does your household mainly use for cooking? | 1.Electricity  2. Liquefied petroleum gas  3. Natural gas  4.Biogas  5.Keresone  6. Charcoal  7. Wood  8. Straw/shrubs/grass  9. Agricultural crop  10. Animal dung  11.No food cooked in household |  |  |
| 212 | Is the cooking usually done in the house, in a separate building, or outdoors? | 1. In the house  2. In a separate building  3. Outdors  4. Others (specify)……………… |  |  |
| 213 | Do you have a separate room which is used as a kitchen? | 1. Yes  0. No |  |  |
| 214 | Who is the owner of the house? | 1. Me 2. Rental 3. Family 4. Relative 5. Others (specify)………….. |  |  |
| 215 | How many rooms in this household are used for sleeping? | -------------------------- |  |  |
| 216 | Main material of the roof of the house? | 1. Natural roofing (no roof, mud, and sod) 2. Rudimentary roofing (rustic mat/plastic shee, reed/bamboo, wood planks,and cardboard) 3. Finished roofing (metal/corrugated iron, wood, calamine/cement, ceramic tiles, roofing shingles) |  |  |
| 217 | Main material of the floor of the house? | 1. Natural floor (Earth/sand, dung) 2. Rudimentary floor (wood planks,and palm/bamboo) 3. Finished floor (parquet or polished wood, vinyl or asphalt strips/ plastic tiles, cement, ceramic tiles, carpet) |  |  |
| 218 | Main material of the wall of the house? | 1. Natural walls (no walls, cane/palm/trunks/bamboo/ree, dirt) 2. Rudimentary walls (bamboo with mud, stone with mud, uncovered adobe, plywood, cardboard, and reused wood ) 3. Finished floor (cement, stone with lime/cement, bricks, cement blocks, covered adobe, wood planks/shingles) |  |  |
| 219 | Does this household own any livestock, herds, other farm animals, or poultry? | 1.Yes  0.No | 222 |  |
| 220 | How many of the following animals does this household own?  IF NONE, RECORD ‘00’.  IF 95 OR MORE, RECORD ‘95’.  IF UNKNOWN, RECORD ‘98’.  a) Milk cows, oxen or bulls?  b) Other cattle?  c) Horses/donkeys/mules?  d) Camels?  e) Goats?  f) Sheep?  g) Chickens/poultry?  h) Beehives? | a) Milk cows, oxen or bulls………  b) Other cattle………………….  c) Horses/donkeys/mules……….  d) Camels……………………..  e) Goats………………………  f) Sheep………………………….  g) Chickens/poultry……………..  h) Beehives……………………. |  |  |
| 221 | Do you have separate rooms for cattle? | 1.Yes  0.No |  |  |
| 222 | Does any member of this household own any agricultural land? | 1.Yes  0. No | 224 |  |
| 223 | How many hectares of agricultural land do members of this household own? | ---------------------------hectares |  |  |
| 224 | Does your household have:   1. Electricity? 2. A radio? 3. A television? 4. A non-mobile phone? 5. A computer? 6. A refrigerator? 7. A table? 8. A chair? 9. A bed with cotton/sponge/spring mattress? 10. An electric mitad? 11. A kereson lamp/pressure lamp? | Yes No   1. Electricity……..1 0 2. Radio…………..1 0 3. Television………1 0 4. Non-mobile phone.1 0 5. Computer…………1 0 6. Refrigerator………1 0 7. Table………………1 0 8. Chair………………1 0 9. Bed with cotton/sponge/spring mattress…………..1 0 10. Electric mitad…….1 0 11. Kereson lamp/pressure lamp……………….1 0 |  |  |
| 225 | Does any member of this household own:  a) A watch?  b) A mobile phone?  c) A bicycle?  d) A motocycles/scooter?  e) An animal drawn cart?  f) A car/truck?  g) A boat with motor?  h) A bajaja? | Yes No a) Watch . . . . . . . . . . 1 0  b) Mobile phone . . . . . 1 0  c) Bicycle. . . . . . . . . . . 1 0  d) Motocycles/scooter. . 1 0  e) Animal drawn cart . . .1 0  f) Car/truck . . . . . . . . . . .1 0  g) Boat with motor . . . . . 1 0  h) Bajaja . . . . . . . ………1 0 |  |  |
| 226 | Does any member of this household have a bank account? | 1. Yes 2. No |  |  |
| 227 | Does any member of this household have a microfinanceaccount? | 1. Yes 2. No |  |  |

**Module III: Reproductive history of study participants**

| S.no | Questions | Response categories | Skip to | Remark |
| --- | --- | --- | --- | --- |
| 301 | What was your age when you were first married? | 998. I don’t know |  |  |
| 302 | What was your age at your first pregnancy? | 998. I don’t know |  |  |
| 303 | Have you ever become a pregnant? | 1. No 2. Yes | 3 |  |
| 304 | If yes for question 303, how many times? | --------------------------- |  |  |
| 305 | Have you ever faced abortion? | 1. No 2. Yes | 307 |  |
| 306 | If yes for question 305, how many times? | ---------------------------- |  |  |
| 307 | Have you ever delivered a child? | 1. No 2. Yes | 311 |  |
| 308 | If yes for question 306, how many times? | ----------------------------- |  |  |
| 309 | How many of them were live births? | ------------------------------ |  |  |
| 310 | How many of them were delivered at home? | --------------------------- |  |  |
| 311 | Infection during the current pregnancy (e.g., UTI, periodontal disease) | 1. No 2. Yes |  |  |
| 312 | Do you have any family history of hypertension? | 1. No 2. Yes | 3 |  |
| 313 | If Yes to Q312, who? | 1. Father 2. Mother 3. Sister 4. Grand mother 5. Other (specify)……………….. |  |  |
| 314 | Have you ever experienced death of fetus or stillbirth (fetal death at or after 28 weeks of pregnancy)? | 0. Yes  1. No  998. I don‘t remember |  |  |
| 315 | Have you ever experienced death of neonate (age less than seven days)? | 0. Yes  1. No  998. I don‘t remember | end |  |
| 316 | If yes to Q 314, how many times? | _____________ |  |  |
| 317 | If yes to Q 314, where was the neonate born? | 1. Home  2. Health institution  3. Other (specify) _________ |  |  |

**Module IV: Maternal health service utilization**

| S.no | Questions | Response categories | Skip to | Remark |
| --- | --- | --- | --- | --- |
| 401 | Was your last pregnancy planned? | 1. Yes  0. No | 405 |  |
| 402 | If ‘No’ to Q401, what was reason? | 1. Lack of modern contraceptives  2. Failure of contraceptive method  3. Others (specify)………………… |  |  |
| 403 | If ‘No’ to Q401, don’t want at all the last pregnancy? | 1. Yes  0. No |  |  |
| 404 | If ‘No’ to Q401, do you want after months of delivery? | 1. Yes  0. No |  |  |
| 405 | Did you face any health problem during your the last pregnancy? | 1. Yes  0. No | 407 |  |
| 406 | If ‘Yes’ to Q 405, what were they?  (Do not read the choices) | 1. Excessive vaginal bleeding 2. A high grade fever 3. Bad smelling vaginal discharge 4. Convulsions 5. Severe headache 6. Face/ hand swelling 7. Persistent vomiting 8. High blood pressure 9. Other (specify)_________ |  |  |
| 407 | Did you visit a health facility during your last pregnancy? | 1. Yes  0. No | 409 |  |
| 408 | If yes to Q 407, reason for visit? | 1. Pregnancy related health problem  2. Health problems not related to pregnancy  3. For antenatal care  4. Other (specify) ------------------ |  |  |
| 409 | Did you obtain ANC visit during your last pregnancy? | 1. Yes  0. No  998. Don’t remember | 428  428 |  |
| 410 | If yes to Q409, how many times did you obtain ANC? | 998.Don’t know/remember | 412 |  |
| 411 | Mention chronologically in how many months did you obtain ANC visits? | 1^st^ ANC ……………….months  2^nd^ ANC ………………months  3^rd^ ANC………………..months  4^th^ ANC……………….months  999. Don’t know/remember |  |  |
| 412 | From where did you receive ANC service? | 1. Government hospital  2. Private hospital  3. Faith based charity ospital  4. Health centre  5. Private clinic  6. Faith based charity clinic  7. Health post  8. Other (specify) ________________ |  |  |
| 413 | Why did you prefer the above stated health facility (Q412)? | 1. Close to my house  2. Competent health worker  3. Other (specify) _________ |  |  |
| 414 | Who provided you ANC in the last pregnancy? | 1. Doctors 2. Nurse 3. Midwifes 4. Health officers 5. Health extension workers 6. Traditional birth attendant 7. Other (specify)___________ |  |  |
| 415 | Did you receive any tetanus toxoid (TT) injection during your last ANC visits? | 1. Yes 2. No   998. Don’t remember |  |  |
| 416 | If ‘Yes’ to Q415, how many times? | __________________ |  |  |
| 417 | Did you receive any laboratory test during your last ANC visits? | 1. Yes  0. No  998. Don’t remember |  |  |
| 418 | If ‘Yes’ to Q417, what tests? | 1= Blood (Blood groups)  2= Blood (Syphilis test)  3= Stool  4= Urine  5= Other (Specify)……………………… |  |  |
| 419 | Did you receive any health message during your last ANC visits? | 1. Yes  0. No  998. Don’t remember | 428  428 |  |
| 420 | If ‘Yes’ to Q419, what messages? | 1= Breastfeeding  2= Danger Signs Maternal  3= Birth Planning  4= Facility Delivery  5= Danger Signs New-born  6= Basic Care New Born  8= Other (Specify)……………………… | 421 |  |
| 421 | Which danger signs of were you informed about?  (Don‘t read the choices) | l= Severe headache  2= Blurry Vision  3= Reduced or absent foetal movement  4= High blood pressure  5= Oedema of the face  6= Oedema of the hands & feet  7= Convulsions  8= Vaginal bleeding  9= Lower abdominal pain  l0=None of above |  |  |
| 422 | Were you informed where to go during danger signs problems stated above (Q421)? | 1. Yes  0. No  998. Do not remember |  |  |
| 423 | Where you informed about where to deliver during your last baby? | 1. Yes  0. No  998. Do not remember | 425  425 |  |
| 424 | If yes to Q 423, where were you recommended to deliver? | 1. Home 2. Health post 3. Health centre 4. Private clinic 5. Faith based charity clinic 6. Government hospital 7. Private hospital 8. Faith based charity hospital 9. Others specify……………… |  |  |
| 425 | Were you informed about who should attend you during delivery? | 1. Yes  0. No  998. Do not remember | 427  427 |  |
| 426 | If ‘Yes’ to Q 425, who was recommended to attend your delivery? | 1. Doctors 2. Nurse/Midwifes/health officers 3. HEWs 4. Traditional birth attendant 5. Other (specify)__________ |  |  |
| 427 | Were you informed to prepare the following items before your delivery date? | 1. Transport  2. Save money  3. Identify blood donor  4. Nothing |  |  |
| 428 | If you didn’t ANC visits, can you tell me the reasons?  (Multiple answer is possible)  (Do not read the choice) | 1.No or little Knowledge about ANC  2. No health problem encountered  3. Health institution is too far from my home  4. Long waiting time  5. Poor handling by health care providers  6. Lack of transportation  7. Lack of time to go to health institution  8. Other (specify)---------------------- |  |  |
| 429 | Did you plan to deliver in health facility? | 1. Yes  0. No  998. Don’t remember | 431 |  |
| 430 | If no to Q429, can you tell me the reasons?  (Multiple answer is possible)  (Do not read the choice) | 1.No or little Knowledge about institutional delivery  2. No health problem encountered  3. Health institution is too far from my home  4. Poor handling by health care providers  5. Lack of transportation  6. Other (specify)---------------------- |  |  |
| 431 | Before your date of delivery for your last pregnancy did you or your family prepared the following? | 1. Transport  2. Save money  3. Identify appropriate health facility  4. Identify blood donor  5. Identify skilled attendant  6. Nothing |  |  |
| 432 | Where did you give birth to your last child? | 1. Home 2. Health post 3. Health centre 4. Private clinic 5. Faith based charity clinic 6. Government hospital 7. Private hospital 8. Faith based charity hospital 9. Others specify……………… | 442 |  |
| 433 | If your last delivery is in health institution, when did you go to the health institution? | 1. At the beginning of labour  2. 6-12 hours after the beginning of labour  3. 13-18 hours after the beginning of labour  4. 19-24 hours after the beginning of labour  5. Other specify __________ |  |  |
| 434 | If your last delivery is in health institution, what was the mode of delivery? | 1. Spontaneous vaginal delivery  2. Instrumental delivery  3. Caesarean section  4. Other (specify) __________ |  |  |
| 435 | Who conducted or assisted you in the last delivery? | 1. Doctors 2. Nurse 3. Midwife 4. Health officer 5. Health extension worker 6. Traditional birth attendant (TBA) 7. Other (specify)___________ |  |  |
| 436 | Did anyone other than health care providers assist you with the delivery of last baby? | 1. Yes 2. No |  |  |
| 437 | If ‘Yes’ to Q436, who assisted you with the delivery?  Anything else?  RECORD ALL RESPONSES | 1. Mother 2. Mother-in law 3. Sister 4. Sister-in law 5. Other member of in-laws home 6. Other member of fathers 7. Other relatives 8. Neighbour 9. Other specify…………………… |  |  |
| 438 | Were any of the following procedures performed at the time of delivery? | 1. Blood transfusion  2. Fluid  3. Injection  4. Episiotomy  5. Received tablet Misoprostol |  |  |
| 439 | If injection provided. What was the injection for?  (Do not read the choice)  RECORD ALL RESPONSES | 1= Speed up labour  2 = Reduce bleeding  3= Stop convulsions  4=Other (Specify)  999=Don’t Know |  |  |
| 440 | For how long were you in labour during the last delivery? | 1. Less than 12 hours  2. 12- 24 hours  3. 25- 36 hours  4. 37 – 48 hours  5. More than 48 hours |  |  |
| 441 | What was the condition of the baby at birth in the last delivery? | 1. Born alive  2. Still birth (born died)  3. Born alive but died immediately |  |  |
| 442 | If responded at home to Q432, why did you prefer to deliver at home?  (Don‘t read the choice) | 1. The labour was short  2. No nearby health facility  3. The service is not available in the nearby health facility  4. Lack of money for transport  5. Poor handling by health professionals  6. Prefer to deliver in the presence of relatives  7. Fear of manipulation (like episiotomy)  8. Lack of privacy in the health institutions  9. I didn‘t know the importance of health facility delivery  10.Opinions of (husband, neighbours, other community members)  11. Other (specify) ____________ |  |  |
| 443 | Have you encountered any health problems during labour in the last delivery? | 1. Yes  0. No  999. I don‘t remember | 456 |  |
| 444 | If yes to Q 443, what were they?  (Do not read the choices) | 1 = Excessive Vaginal bleeding  2 = Foul smelling discharge  3 = High grade fever  4 = Baby’s hand or feet come first  5= Baby bad position/ mal presentation  6= Prolong labour (> l2 Hours)  7= Retained placenta/placental expelled late  8 = Torn uterus  9= Prolapsed cord  10 = Cord around the neck  11= Convulsions  l2 = Perineal tear  l3= None of above | 456 |  |
| 445 | Did you seek any care or treatment for any of these/this complication(s)?  Probe: Any care/treatment receive at home | 1. Yes 2. No | 456 |  |
| 446 | If ‘Yes’ to Q445, where did you seek care/treatment for these/this complication(s)?  RECORD ONLY ONE ANSWER- WHERE WOMAN OR FAMILY FIRST SOUGIIT CARE/TREATMENT | 1=Home  2=Health post  3=Health centre  4= Private clinic  5=Faith based charity clinic  6=Government hospital  7=Private hospital  8=Faith based charity hospital  9=Others specify……………… |  |  |
| 447 | If ‘Yes’ to Q445, who provided this care/treatment?  RECORD ONLY ONE ANSWER | 1=Doctors  2=Nurse  3=Midwife  4=Health offer  5=Health extension worker  6=Traditional birth attendant (TBA)  7=Family  8=Relative  9=Neighbour  10=Other (specify)___________ |  |  |
| 448 | Were you referred to any other place for care/treatment of complication(s)?  PROBE. CARE INSIDE/OUTSIDE THE HOME? | 1. Yes 2. No | 451 |  |
| 449 | If ‘Yes’ to Q448, where were you referred? | 1. Health post 2. Health centre 3. Hospital 4. Prayer 5. Other specify…………………….. |  |  |
| 450 | Did they tell you the reasons for referral? | 1. Yes 2. No | 455 |  |
| 451 | If ‘Yes’ to Q449, why were you told to seek treatment at this than other place?  RECORD ALL RESPONSES | 1 = No surgery equipment  2 = High blood pressure  3 = For better treatment  4 = Doctor unavailable  5 = No arrangement for blood transfusion  6 = Baby’s upward position  7 = Some part of baby came out  8 =Baby Stool in womb  9 = cervix did not open  10 = Other specify  999 = Don’t know |  |  |
| 452 | Did you go to this place? | 1. Yes 2. No   999. Don’t remember | 455 |  |
| 453 | If ‘Yes’ to Q452, how long did you wait to seek care for this complication? | _______________ |  |  |
| 454 | If ‘Yes’ to Q449, did anybody call you after you want to the referred place, to see how you were doing? | 1. Yes 2. No   999. Don’t remember |  |  |
| 455 | Why did you not go to the referred place? | 1 = Woman didn’t think necessary  2 = Husband /family didn’t think necessary  3 = Facility too far  4 = No transport  5 = No child care  6 = too expensive  7 = Services are poor quality  8 = Didn’t know where to go  9 = No time to go  10 = Money not easily available  I l = Other (Specify)  99 = Don’t know |  |  |
| 456 | Did you use the transport you identified during pregnancy? | 1. Yes 2. No |  |  |
| 457 | Did you use the money that you saved during pregnancy? | 1. Yes 2. No |  |  |
| 458 | Have you encountered any health problems during the first 6 weeks of post-partum? | 1. Yes  0. No  999. I don‘t remember | 460 |  |
| 459 | If ‘Yes’ to Q 455, what were they?  (Do not read the choices) | 1 = Excessive vaginal bleeding  2 = Foul smelling discharge  3 = High fever  4 = Inverted nipples  5 = Tetanus  6 = Retained placenta  7 = Severe abdominal pain  8 = Convulsions  9 = Engorged breasts  10 = None of above |  |  |
| 460 | Did you get medical check-up or PNC after your last delivery within 42 days? | 1. Yes  0. No  999. I don‘t remember |  |  |
| 461 | If ‘Yes’ to Q461, when did you get it for the first time after delivery? | 1. After ______ hours  2. After ______ days  3. After ______ weeks |  |  |
| 462 | If ‘Yes’ to Q461, how many times did you obtain PNC within 42 days? | 998.Don’t know/remember |  |  |
| 463 | Where did you get the check-up for the first time? | 1= Home  2=Health post  3=Health centre  4=Hospital  5=Others specify……………… |  |  |
| 464 | What additional services did you get? | 1. Child vaccination  2. Family planning methods  3. Counselling about breast feeding  4. Others specify ____________ |  |  |
| 465 | From where did you receive PNC service? | 1. Government hospital  2. Private hospital  3. Faith based charity ospital  4. Health centre  5. Private clinic  6. Faith based charity clinic  7. Health post  8. Other (specify) ________________ |  |  |
| 466 | Who provided you PNC in the last childbirth? | 1=Doctors  2=Nurse  3=Midwifes  4=Health officers  5=Health extension workers  6=Traditional birth attendant  7=Other (specify)___________ |  |  |
| 467 | If you didn’t PNC visits, can you tell me the reasons?  (Multiple answer is possible)  (Do not read the choice) | 1.No or little Knowledge about ANC  2. No health problem encountered  3. Health institution is too far from my home  4. Long waiting time  5. Poor handling by health care providers  6. Lack of transportation  7. Lack of time to go to health institution  8. Other (specify)---------------------- |  |  |
| 468 | What was your husband‘s attitude towards MHSU? | 1. Positive  2. Negative  3. Don‘t know |  |  |
| 469 | Who is the decision maker in your HH to seek care from modern health institution? | 1. Both myself and my husband  2. My husband  3. My self  4. Other (specify) _____________ |  |  |
| 470 | Is road accessible to near health facility? | 1. Yes 2. No |  |  |
| 471 | Have you taken model household training? | 1. Yes 2. No |  |  |

**Module V: Knowledge and attitudes of study participants towards MHSU**

**1. Knowledge of study participants towards MHSU**

| S.no | Questions | Response categories | Skip to | Remark |
| --- | --- | --- | --- | --- |
|  | **Knowledge questions for ANC** |  |  |  |
| 501 | Do you know about the ANC service? | 1. 0. No   Yes 1. Yes | 503 |  |
| 502 | If yes to Q 501, is ANC important for mothers and fetus positive health outcome? | 1. No 2. Yes |  |  |
| 503 | When does the first ANC visit is recommended? | 1. Greater than 16 weeks 2. Less than 16 weeks |  |  |
| 504 | Do you know total number of ANC visits recommended for the pregrant women throughout her preganancy period? | 1. Less than 4 2. Four and more |  |  |
| 505 | Where is the appropriate place for the first ANC visit? | 1. Health post 2. Helth center or hospital |  |  |
| 506 | Does a pregnant woman need to undergo the laboratory test during her ANC visit? | 1. No 2. Yes |  |  |
| 507 | Does a pregnant woman need to be provided the essential drugs during her ANC visit? | 1. No 2. Yes |  |  |
| 508 | Does a pregnant woman need to be provided the counseling during her ANC visit? | 1. No 2. Yes |  |  |
|  | **Knowledge questions for institutional delivery** |  |  |  |
| 509 | Do you know about the institutional delivery service? | 1. No 2. Yes |  |  |
| 510 | If yes to Q 501, is institutional delivery care important for mothers and newborn positive outcome? | 1. No 2. Yes |  |  |
| 511 | Which woman is recommended to give birth at HFs? | 1. High risk 2. All |  |  |
| 512 | Which place is safe for child delivery? | 1. Home 2. HFs |  |  |
| 513 | Which provider is skilled for child delivery? | 1. TBA, relative, friend 2. Health professional |  |  |
| 514 | Does a woman die due to the complications happen during child birth? | 1. No 2. Yes |  |  |
| 515 | Does a SBA at HFs can prevent maternal deaths that happen during the child birth? | 1. No 2. Yes |  |  |
|  | **Knowledge questions for PNC** |  |  |  |
| 516 | Do you know about the PNC service? | 1. No 2. Yes |  |  |
| 517 | If yes to Q 501, is PNC important for mothers and neonetus positive health outcome? | 1. No 2. Yes |  |  |
| 518 | Do you know total number of PNC visits recommended for the women throughout her pospartum period? | 1. Less than 3 2. Three and more |  |  |
| 519 | Where is the appropriate place for the first PNC visit? | 1. Health post 2. Helth center or hospital |  |  |
| 520 | Does a pregnant woman need to be provided the counseling during her PNC visit? | 1. No 2. Yes |  |  |
| 521 | Does a woman die due to the complications happen during postpartum period? | 1. No 2. Yes |  |  |
| 522 | Does a PNC at HFs can prevent maternal deaths that happen during the postpartum period? | 1. No 2. Yes |  |  |

1. **Attitude of study participants towards MHSU**

**Tool to assess client attitude towards MHSU**

1-Strongly disagree; 2- Disagree; 3- Neutral; 4- Agree; 5- Strongly agree

| S.no | Questions | 1 | 2 | 3 | 4 | 5 |
| --- | --- | --- | --- | --- | --- | --- |
|  | **Attitude questions for ANC** |  |  |  |  |  |
| 523 | Your attitude about the ANC is important for mothers' positive health outcomes? |  |  |  |  |  |
| 524 | Your attitude about the first ANC visit should be obtained in less than 16 weeks? |  |  |  |  |  |
| 525 | Your attitude about the four ANC visits is adequate for uncomplicated pregnancy? |  |  |  |  |  |
| 526 | Your attitude about the health post is an inappropriate place for the first ANC visit? |  |  |  |  |  |
| 527 | Your attitude about the pregnant woman needs to use the laboratory test during her ANC visit? |  |  |  |  |  |
| 528 | Your attitude about the pregnant woman needs to be providing the essential drugs during her ANC visits? |  |  |  |  |  |
| 529 | Your attitude about the pregnant woman needs to be providing counsel during her ANC visits? |  |  |  |  |  |
| 530 | Your attitude about the women's reasons not to go to a health facility for ANC is mainly because of high opportunity cost? |  |  |  |  |  |
| 531 | Your attitude about the women's reasons not to go to a health facility for ANC because HCPs don't treat them respectfully? |  |  |  |  |  |
|  | **Attitude questions for institutional delivery** |  |  |  |  |  |
| 532 | Your attitude about institutional delivery care is important for mothers’ positive health outcomes? |  |  |  |  |  |
| 533 | Your attitude about all women is recommended to give birth at HFs? |  |  |  |  |  |
| 534 | Your attitude about the HFs is safe for child delivery? |  |  |  |  |  |
| 535 | Your attitude about the HCPs being skilled professionals for child delivery? |  |  |  |  |  |
| 536 | Your attitude about the woman dying due to the complications happens during childbirth? |  |  |  |  |  |
| 537 | Your attitude about the SBA at HFs can prevent maternal deaths that happen during childbirth? |  |  |  |  |  |
| 538 | Your attitude about the importance of having a plan on possible pregnancy complications? |  |  |  |  |  |
| 539 | Your attitude about the delivery being attended by male HCPs isn’t shameful? |  |  |  |  |  |
| 540 | Your attitude about the delivery on the delivery bed isn't shameful in the labour ward? |  |  |  |  |  |
| 541 | Your attitude about the women doesn’t go to a health facility for delivery is due to high opportunity cost? |  |  |  |  |  |
| 542 | Your attitude about the women doesn’t go to a health facility for delivery due to HCPs doesn’t treat them respectfully? |  |  |  |  |  |
|  | **Attitude questions for PNC** |  |  |  |  |  |
| 543 | Your attitude about the PNC is important for mothers’ positive health outcomes? |  |  |  |  |  |
| 544 | Your attitude about the 3 PNC visits is adequate for the women with uncomplicated delivery? |  |  |  |  |  |
| 545 | Your attitude about the health post is an inappropriate place for the first PNC? |  |  |  |  |  |
| 546 | Your attitude about the delivered woman needs to be provided the counsel during her PNC visit? |  |  |  |  |  |
| 547 | Your attitude about the woman dying due to the complications happens during the postpartum period? |  |  |  |  |  |
| 548 | Your attitude about the PNC visits in HFs can prevent maternal deaths that happen during the postpartum period? |  |  |  |  |  |
| 549 | Your attitude about the women doesn’t go to a health facility for PNC is due to high opportunity cost? |  |  |  |  |  |
| 550 | Your attitude about the women doesn’t go to a health facility for PNC is due to HCPs don’t treat them respectfully? |  |  |  |  |  |

**Module VI: Socio-cultural predictors**

| S.no | Questions | Response categories | Skip to | Remark |
| --- | --- | --- | --- | --- |
| 601 | Can you tell me any socio-cultural beliefs that affect MHSU in your community?  (Do not read the choices)  RECORD ALL RESPONSES | 1. Availability and influence of traditional healers  2. Availability and influence of spiritual healers   1. Availability and influence of traditional birth attendants 2. Holy water 3. Fear of using MHS 4. Peer influence 5. Knowledge of community towards MHSU 6. Husband influence 7. Father and mother in law influence 8. Other (specify)…………. |  |  |

**Model VII: Perceived quality of MHSU**

**Tool to assess client perceived quality of antenatal care**

1-Very poor; 2- Poor; 3- Neutral; 4- Good; 5- Very good

|  | **ITEMS** | 1 | 2 | 3 | 4 | 5 |
| --- | --- | --- | --- | --- | --- | --- |
| 1 | Your perception about the friendliness shown towards you by the HCPs in the ANC |  |  |  |  |  |
| 2 | Your perception about the patience shown towards you when you did not cooperate with HCPs |  |  |  |  |  |
| 3 | Your perception about the promptness of the attention given by the HCPs when you needed it |  |  |  |  |  |
| 4 | Your perception about the enough time with HCPs during ANC visit |  |  |  |  |  |
| 5 | Your perception about the way your privacy was maintained by the HCPs in the ANC room |  |  |  |  |  |
| 6 | Your perception about the willingness of the HCPs to discuss about your concerns |  |  |  |  |  |
| 7 | Your perception about the your involvement in decision making |  |  |  |  |  |
| 8 | Your perception about the fairness of waiting time in the ANC visit |  |  |  |  |  |
| 9 | Your perception about the overall cleanliness of the HF |  |  |  |  |  |
| 10 | Your perception about the help you received from the health care workers to take care of fetus and yourself (ex: maintaining your cleanliness) |  |  |  |  |  |
| 11 | Your perception about the adequacy of information given to you on ANC benefits |  |  |  |  |  |
| 12 | Your perception about the adequacy of information given on expected date of delivery and gestational age |  |  |  |  |  |
| 13 | Your perception about the adequacy of information given to you to identify danger signals |  |  |  |  |  |
| 14 | Your perception about the skills of the HCPs to identify and manage health issues |  |  |  |  |  |
| 15 | Your perception about adequacy of information received to clarify any issues you had |  |  |  |  |  |
| 16 | Your perception about the Cleanliness of the ANC room |  |  |  |  |  |
| 17 | Your perception about adequacy of information received on the nutrition |  |  |  |  |  |
| 18 | Your perception about the information on Tetanus toixed vacination |  |  |  |  |  |
| 19 | Your perception about the information on blood test |  |  |  |  |  |
| 20 | Your perception about the information on urine test |  |  |  |  |  |
| 21 | Your perception about the information on weight, height measurement |  |  |  |  |  |
| 22 | Your perception about the information and blood pressure measurement |  |  |  |  |  |
| 23 | Your perception about an appointment clarity |  |  |  |  |  |

**Tool to assess mother perceived quality of institutional delivery care**

1-Strongly disagree; 2- Disagree; 3- Neutral; 4- Agree; 5- Strongly agree

| S.no | Questions | 1 | 2 | 3 | 4 | 5 |
| --- | --- | --- | --- | --- | --- | --- |
| 1 | I think there is good waiting area in the nearby health facility |  |  |  |  |  |
| 2 | I think there is good maternity ward’s toilets in the nearby health facility |  |  |  |  |  |
| 3 | Hand washing and shower is available in the nearby health facility |  |  |  |  |  |
| 4 | Infrastructures like electricity, and water is available in the nearby health facility |  |  |  |  |  |
| 5 | It is easy to get maternity ward starting from the gate |  |  |  |  |  |
| 6 | The health care providers in the nearby clinic are good at labor and delivery care |  |  |  |  |  |
| 7 | The health care providers in the nearby clinic are good at newborn care |  |  |  |  |  |
| 8 | The health care providers in the nearby clinic are good at pain management |  |  |  |  |  |
|  | | | | | | |
| 9 | How long does it take to reach to the nearby health facility on foot? | ________hrs | | | |  |
| 10 | Prompt transport service is available from home to the nearby health facility | 1. Yes  0. No | | | |  |
| 11 | Delivery service charge in the nearby health facility | 1. High  2. Moderate  3. Low | | | |  |

**Tool to assess client perceived quality of institutional postnatal care**

1-Very poor; 2- Poor; 3- Neutral; 4- Good; 5- Very good

|  | **ITEMS** | 1 | 2 | 3 | 4 | 5 |
| --- | --- | --- | --- | --- | --- | --- |
| 1 | Your perception about the friendliness shown towards you by the HCPs in the postnatal ward |  |  |  |  |  |
| 2 | Your perception about the patience shown towards you when you did not cooperate with HCPs |  |  |  |  |  |
| 3 | Your perception about the promptness of the attention given by the HCPs when you needed it |  |  |  |  |  |
| 4 | Your perception about the availability of pain relief during the postpartum period |  |  |  |  |  |
| 5 | Your perception about the way your privacy was respected by the HCPs in the postnatal ward |  |  |  |  |  |
| 6 | Your perception about the willingness of the HCPs to discuss about your concerns |  |  |  |  |  |
| 7 | Your perception about the way HCPs treated your family members |  |  |  |  |  |
| 8 | Your perception about the help given for the initiation of breast feeding in the labour room |  |  |  |  |  |
| 9 | Your perception about the help you received from the HCPs to take care of your baby |  |  |  |  |  |
| 10 | Your perception about the help you received from the health care workers to take care of yourself (ex: maintaining your cleanliness) |  |  |  |  |  |
| 11 | Your perception about the adequacy of information given to you on taking care of the baby |  |  |  |  |  |
| 12 | Your perception about the adequacy of information given on proper method of breast feeding |  |  |  |  |  |
| 13 | Your perception about the adequacy of information to identify danger signals following delivery, for the mother & the baby |  |  |  |  |  |
| 14 | Your perception about the skills of the HCPs to identify and manage health issues of your baby |  |  |  |  |  |
| 15 | Your perception about the skills of the HCPs to identify and manage health issues in relation to you |  |  |  |  |  |
| 16 | Your perception about adequacy of information received to clarify any issues you had |  |  |  |  |  |
| 17 | Your perception about the Cleanliness of the ward |  |  |  |  |  |
| 18 | Your perception about the Cleanliness of the toilets & washrooms |  |  |  |  |  |
| 19 | Your perception about adequacy of space in the postnatal ward |  |  |  |  |  |
| 20 | Your perception about the availability of adequate facilities in the ward in relation to the number of patients |  |  |  |  |  |
| 21 | Your perception about adequacy of delivery beds in the labour room |  |  |  |  |  |
| 22 | Your perception about the availability of adequate numbers of HCPs to assist you |  |  |  |  |  |
| 23 | Your perception about the ability to get some rest in the postnatal ward (without the interferences such as light, noise, ward activities) |  |  |  |  |  |

**Module IIX: Knowledge of ODS during pregnancy, childbirth and postpartum periods**

| S.no | Questions | Response categories | Skip to | Remark |
| --- | --- | --- | --- | --- |
| 801 | Please tell me what complications may occur during pregnancy that needs rnedical care?  (Do not read the choices)  Anything else?  RECORD ALL RESPONSES | l= Severe headache  2= Blurry vision  3= Reduced or absent foetal movement  4= High blood pressure  5= Oedema of the face  6= Oedema of the hands & feet  7= Convulsions  8= Vaginal bleeding  9= Lower abdominal pain  l0=None of above |  |  |
| 802 | Now, please tell me what are the complications in women during child birth that need medical treatment?  (Do not read the choices)  Anything else?  RECORD ALL RESPONSES | 1 = Excessive vaginal bleeding  2 = Foul smelling discharge  3 = High grade fever  4 = Baby's hand or feet come first  5= Baby bad position/ mal presentation  6= Prolong labour (> l2 Hours)  7= Retained placenta  8 =Torn uterus  9= Prolapsed cord  10 = Cord around the neck  11= Convulsions  l2 = Perineal Tear  l3= None of above |  |  |
| 803 | Now, please tell me what are the complications in women immediately child birth that need medical treatment?  (Do not read the choices)  Anything else?  RECORD ALL RESPONSES | 1 = Excessive vaginal bleeding  2 = Foul smelling discharge  3 = High fever  4 = Inverted nipples  5 = Tetanus  6 = Retained placenta  7 = Severe abdominal pain  8 = Convulsions  9 = Engorged breasts  10 = None of above |  |  |

**Module IX: Practices of BPCR**

| S.no | Questions | Response categories | Skip to | Remark |
| --- | --- | --- | --- | --- |
| 901 | During pregnancy and before the delivery, did you or your family make any plans for the birth? | l= Yes  0= No  999= Don't Remember | 906 |  |
| 902 | If yes to Q901, which plans did you or your family makes for the birth of (NAME)?  (Do not read the choices)  Anything else?  RECORD ALL RESPONSES | l= Identify proper closer HFs for childbirth  2= Identify SBA for deliver  3= Save money and material resources  4= Identify proper transport for delivery  5= Identify and fixing the compatible blood group givers |  |  |
| 903 | If identified facility for delivery. Where did you plan to give birth to (NAME)?  (Do not read the choices) | 1. Home 2. Health post 3. Health centre 4. Hospital 5. Others specify……………… |  |  |
| 904 | If identified provider for delivery. Who did you select as the birth attendant?  (Do not read the choices) | 1. Doctors 2. Nurse/Midwifes 3. Health extension workers 4. Traditional birth attendant 5. Other (specify)___________ |  |  |
| 905 | If identified transport. Did you use the transport you identified during pregnancy? | 1. Yes 2. No |  |  |
| 906 | If saved money. Did you use the money that you saved during pregnancy? | 1. Yes 2. No |  |  |

**Thank you for your participation!!!**
