# Supplementary material for: Addressing abortion through individual and community-level determinants: Evidence from Southern Ethiopia
Source: PLoS One. 2026 Jun 17;21(6):e0349603. doi: 10.1371/journal.pone.0349603 (PMC13274891; doi:10.1371/journal.pone.0349603)
Supplement: Attachment — Submitted filename: Pone-D-25-03111_reviewer.docx [file pone.0349603.s005.docx]

**Comments of Manuscript 03111**

**Methodology**

Study design

“The study rationales receiving high–quality ANC interacts with the updated WHO recommendations to improve the use of SBA and facility deliveries”. This study has not been explicitly reported in the study findings. If I have missed please explain where the quality and quantity of ANC services combine to give interactive effect on health care delivery service utilization

Sampling

How was 675 EAs chosen? The basis for choosing exactly that number. The second stage was EA cluster. Why was not design effect not applied?

“variables coded as 1 (good QANC) if the pregnant women received all seven essential ANC components at least once and 2 (poor QANC) otherwise” What is the justification of this stringent criteria of all/none (can it not be in some cases one of the components were not fulfilled because it was not indicated?) how do you justify it.? The good QANC is only 21.6% probably need to be rechecked using sensitivity analysis using (0-7) score or different cutoffs to test the robustness

ANC visit categorization is (1-3) overlaps between inadequate and almost adequate (WHO recommended 4). Justify if three visits are considered in adequate. What percentage of SBA codded 1 had FD code 1? This looks like overlapping variables.

**Analysis:**

How is missing data handled? (complete case analysis? number excluded? etc).

Mention about potential bias occurring out of from excluding flooded clusters.

WHO recommends 8 visits just to ensure quality antenatal checkups. If ultimately we have to rely on QANC as valid indicator to ensure proper delivery service utilization then why 8 ANC is taken as a second key exposure when QNC means better indicator whether 4 six or 8 Visits are done.

Moreover, Bangladesh’s national guidelines still promote four or more ANC visits [13], then we know low quality service utilization is because of national policy. Only 47% observed in latest survey [12]. Then, is it not better to classify 8 ANC visit by less than 4 and 4 and more?

A woman may attend eight or more visits but still miss critical components such as blood pressure monitoring, essential tests, or comprehensive counseling on danger signs. How do you justify this? Do you consider 8 visits ensure quality ANC? Won’t it be better to consider both variables as an interactive variables to predict better service utilization for delivery?

**Results:**

Line 246-266, if Chi-square test is used to test the association in bivariate tables, how is it significantly higher or lower proportion? Is this proportion test?

Line 257 “The rates were significantly….” Have you calculated rates of SD and FD or it is proportion like you have mentioned in the previous line?

**Discussion**

Line 365 no association of danger signs and SBA and FD. What could be the reasons for Nigerian women with danger sign not turning up at safe delivery care facilities/manpower in this context? When actually they need more specialized care compared to normal women? Not discussed in detail.
