# Supplementary material for: Addressing abortion through individual and community-level determinants: Evidence from Southern Ethiopia
Source: PLoS One. 2026 Jun 17;21(6):e0349603. doi: 10.1371/journal.pone.0349603 (PMC13274891; doi:10.1371/journal.pone.0349603)
Supplement: Attachment — Submitted filename: Response to Reviewers.docx [file pone.0349603.s008.docx]

***Point-by-Point Response to Reviewers’ and Editor Comments***

**Title:** *Addressing Abortion through Individual and Community-Level Determinants: Evidence from Southern Ethiopia*

**Manuscript ID:** *PONE-D-25-55056*

**Journal Requirements**

**Comment 1.** Please ensure that your manuscript meets PLOS ONE's style requirements, including those for file naming. The PLOS ONE style templates can be found at  <https://journals.plos.org/plosone/s/file?id=wjVg/PLOSOne_formatting_sample_main_body.pdf> and  <https://journals.plos.org/plosone/s/file?id=ba62/PLOSOne_formatting_sample_title_authors_affiliations.pdf>
**Authors’ response:** Thank you for this comment. We have reviewed the manuscript and revised it to ensure full compliance with the PLOS ONE formatting and style requirements, including file naming, manuscript structure, and the presentation of the title page and main text according to the journal’s templates.

**Comment 2.** Thank you for stating the following in the Acknowledgments Section of your manuscript: “This study was supported by Nestlé Foundation with grant number of HUHS/021/2016. The funders had no role in the design and conduct of the study; collection, management, analysis, and interpretation of the data; preparation, review, or approval of the manuscript; and decision to submit the manuscript for publication.”

We note that you have provided funding information that is not currently declared in your Funding Statement. However, funding information should not appear in the Acknowledgments section or other areas of your manuscript. We will only publish funding information present in the Funding Statement section of the online submission form.

Please remove any funding-related text from the manuscript and let us know how you would like to update your Funding Statement. Currently, your Funding Statement reads as follows:  “This study was supported by Nestlé Foundation with grant number of HUHS/021/2016. The funders had no role in the design and conduct of the study; collection, management, analysis, and interpretation of the data; preparation, review, or approval of the manuscript; and decision to submit the manuscript for publication.” Please include your amended statements within your cover letter; we will change the online submission form on your behalf.

**Authors’ response:** Thank you for this clarification. In line with the journal’s guidance, we have removed the funding information from the Acknowledgments section. The correct funding source is the Sidama Region President Office (Grant No. SPO/123/16), and this information should appear only in the Funding Statement of the submission system. The funder had no role in the study design, data collection, analysis, interpretation, manuscript preparation, or the decision to submit the manuscript.

**Comment 3.** Please include a separate caption for each figure in your manuscript.

**Authors’ response:** Thank you for this comment. We have revised the manuscript to ensure that each figure now includes a separate caption, in accordance with PLOS ONE formatting requirements.

**Comment 4.** Thank you for providing your underlying data as Supporting Information. We note that the data set contains text or data that is not in English. Please note that PLOS is an English-language publisher, so we require data sets to be provided in English as well. Please upload an English-language version of your data set. This will also allow us to determine if your data follows PLOS standards per our Data Availability policy here: <https://journals.plos.org/plosone/s/data-availability>

**Authors’ response:** Thank you for this comment. We have carefully translated the dataset into English and uploaded the revised English-language version as Supporting Information to fully comply with PLOS ONE’s Data Availability policy.

**Comment 5.** If the reviewer comments include a recommendation to cite specific previously published works, please review and evaluate these publications to determine whether they are relevant and should be cited. There is no requirement to cite these works unless the editor has indicated otherwise.

**Authors’ response:** Thank you for the guidance. The reviewers did not recommend any specific references, and after careful consideration, we did not add any additional citations to the manuscript.

**Reviewer 1**

**Comment 1:** The overall idea of multimodal analysis is awe-inspiring but not fully explored. And the study would be attractive if it were concentrated on a specific abortion (induced abortion). At least 15% of pregnancies ended up in abortion, the majority caused by chromosomal abnormality, where the community level has no role in determining their occurrence.

**Authors’ response:** Thank you for this important comment. We acknowledge that abortions can result from diverse causes, including biological factors such as chromosomal abnormalities, which primarily account for early spontaneous abortions and are not influenced by community-level factors. However, our study examines abortions as reported by women in the community, encompassing both induced and spontaneous cases, to understand the broader social and environmental determinants of reproductive outcomes.

In settings like Hawela Lida District, a substantial proportion of abortions are likely influenced by preventable or socially mediated factors, including unintended pregnancies, limited autonomy in decision-making, restricted access to healthcare, and sociocultural pressures. Community-level factors such as rural residence, proximity to health facilities, and exposure to mass media - can shape reproductive behaviors and access to family planning, thereby affecting the occurrence of abortions that are socially or behaviorally mediated.

While biological causes contribute to some spontaneous abortions, our findings show that many cases are influenced by factors that can be addressed through public health interventions. We have revised the manuscript to clearly acknowledge the limits of community-level influence on biologically determined abortions while highlighting the relevance of multilevel determinants for most cases observed in this population.

**Methodology
Comment 2:** In line 179. Parity refers to the number of live births, which is not necessarily the number of live births. Even a stillbirth was a parity.

**Authors’ response:** Thank you for highlighting this point. We acknowledge that, by definition, parity refers to the number of pregnancies reaching a viable gestational age, including live births, stillbirths, and sometimes multiple births. In our study, we carefully measured parity as the total number of pregnancies that progressed to ≥28 weeks gestation, thereby including both live births and stillbirths. We have clarified this definition in the Methods section to accurately reflect standard obstetric terminology and ensure consistency with established reporting practices.

**Statistical analysis**

**Comment 3:** About the pregnancy status, it would be better to use the planned delivery as a reference.
**Authors’ response:** Thank you for this suggestion. We agree that using planned pregnancies as the reference category provides clearer interpretability of the odds ratios and aligns with common epidemiological practice. Accordingly, we have updated our multivariable analysis so that planned pregnancies now serve as the reference group, and the results have been revised in Table 3 and throughout the text to reflect this change.

**Comment 4:** About the Woman's decision-making power, it would be better to use Autonomous as a reference.

**Authors’ response:** Thank you for this recommendation. We agree that using autonomous women as the reference category provides clearer interpretability and aligns with standard analytical practice. Accordingly, we have updated our multivariable analysis so that autonomous women now serve as the reference group. All relevant results in Table 3 and throughout the manuscript have been revised to reflect this change.

**Comment 5:** In community-level distance to nearest health facility, it would be better to use not a big problem as a reference.

**Authors’ response:** Thank you for this suggestion. We agree that using “not a big problem” as the reference category for community-level distance to the nearest health facility enhances interpretability of the results. We have updated our multivariable analysis accordingly, and all relevant results in Table 3 and the text have been revised to reflect this change.

**Comment 6:** Variables with p < 0.25 in bivariable analysis or established clinical relevance were included in the multivariable model. The P <0.25 could increase the confounders. I would suggest taking P <0.1 at bivariate analysis to put them in a hierarchical multimodal approach.

**Authors’ response:** Thank you for your valuable suggestion. We have updated the Statistical Analysis section to reflect your recommendation, clarifying that variables with p < 0.10 at the bivariable stage or with established clinical relevance were included in the multivariable model. Upon review, we found that all variables in our analysis already met this threshold, ensuring consistency with a hierarchical multimodal approach.

**Comment 7:** Operational definition should be included to address the following: Community-level mass media use, wealth quintile needs operational definitions and obstetric danger signs during pregnancy

**Authors’ response:** Thank you for the comment. We have added operational definitions for the requested variables: community-level mass media use, wealth quintile, and obstetric danger signs. These are now included in the Methods section.

**Sampling procedure**

**Comment 8:** The sampling procedure would be informative if presented in a plotted figure.

**Authors’ response:** Thank you for this suggestion. We have now created a flowchart illustrating the multistage sampling procedure, from kebele selection to household and participant inclusion. This figure has been added as Figure 2 in the revised manuscript to improve clarity and reader comprehension.

**Eligibility Criteria**

**Comment 9:** Eligibility Criteria: if a resident stays for at least six months and reports a pregnancy that ended in abortion within the past year, this criterion does not necessarily require the abortion to occur within the district. To explain the community level determinants, it should reside for at least one year.

**Authors’ response:** We appreciate this insightful comment. In response, we have updated the eligibility criteria so that women must have resided in Hawela Lida District for at least one year and report a pregnancy that ended in abortion within that period. This modification ensures that all participants have sufficient exposure to the community context, thereby strengthening the validity of our assessment of community-level determinants.

**Ethical Considerations**

**Comment 10:** The reference number should be included.

**Authors’ response:** Thank you for this comment. We have now included the ethical approval reference number in the manuscript. Ethical approval was obtained from the Institutional Review Board of the College of Medicine and Health Sciences, Hawassa University (Ref: /IRB/027/17).

**Results
Comment 11.** The results stated that at the community level, rural residence and high community-level mass media exposure was linked with higher odds of abortion was associated with increased odds of abortion. The high community-level mass media exposure was probably in urban areas, and this contradiction needs to be clearly addressed.

**Authors’ response:** We appreciate the reviewer’s careful observation. Upon review, we realized that the reference was mistakenly assigned in the original manuscript. This has now been corrected to accurately reflect the appropriate source, ensuring clarity and consistency in the text.

**Comment 12.** A table should be prepared for multilevel logistic regression analysis.

**Authors’ response:** We sincerely thank the reviewer for this constructive suggestion. In response, we have created a comprehensive table presenting the results of the multilevel logistic regression analysis, incorporating both individual- and community-level determinants, along with the corresponding adjusted odds ratios and 95% confidence intervals. This table has been added to the revised manuscript to enhance clarity and facilitate interpretation of our findings.

**Reviewer 2**

**General comment:** This study addresses gap by examining a qualitative and quantity of ANC in skilled delivery utilization care thereby contributing to the maternal health literature in Bangladesh and similar LMIC settings. and appropriate multilevel modeling techniques. There are minor queries and issues regarding the definition of outcome and study variable's definition and how does this impact to the primary findings of the study. Please see the attached document for full detail.

**Authors’ response:** We thank the reviewer for acknowledging the significance of our study and the appropriateness of the multilevel modeling approach. We have carefully reviewed all the minor queries regarding outcome definitions and variable operationalization and have addressed them in the revised manuscript. These clarifications do not alter the primary findings but strengthen the transparency and interpretability of our results.

**Comment 1:** The study rationales receiving high–quality ANC interacts with the updated WHO recommendations to improve the use of SBA and facility deliveries”. This study has not been explicitly reported in the study findings. If I have missed please explain where the quality and quantity of ANC services combine to give interactive effect on health care delivery service utilization.

**Authors’ response:** We thank the reviewer for this comment. Our study focuses on abortion and its determinants; issues related to ANC quality or facility deliveries are beyond its scope. The manuscript has been revised to clarify this focus.

**Comment 2:** How was 675 EAs chosen? The basis for choosing exactly that number. The second stage was EA cluster. Why was not design effect not applied? “variables coded as 1 (good QANC) if the pregnant women received all seven essential ANC components at least once and 2 (poor QANC) otherwise” What is the justification of this stringent criteria of all/none (can it not be in some cases one of the components were not fulfilled because it was not indicated?) how do you justify it.? The good QANC is only 21.6% probably need to be rechecked using sensitivity analysis using (0-7) score or different cutoffs to test the robustness. ANC visit categorization is (1-3) overlaps between inadequate and almost adequate (WHO recommended 4). Justify if three visits are considered in adequate. What percentage of SBA codded 1 had FD code 1? This looks like overlapping variables.

**Authors’ response:** We appreciate the reviewer’s detailed feedback. However, our study focuses on abortion and its determinants, not ANC quality or skilled birth attendance. The points raised regarding ANC components, EA selection, design effect, and variable coding pertain to maternal care studies, which are outside the scope of our current analysis. We have clarified this focus throughout the manuscript to avoid confusion.

**Comment 3:** How is missing data handled? (complete case analysis? number excluded? etc). Mention about potential bias occurring out of from excluding flooded clusters. WHO recommends 8 visits just to ensure quality antenatal checkups. If ultimately, we have to rely on QANC as valid indicator to ensure proper delivery service utilization then why 8 ANC is taken as a second key exposure when QNC means better indicator whether 4 six or 8 Visits are done. Moreover, Bangladesh’s national guidelines still promote four or more ANC visits [13], then we know low quality service utilization is because of national policy. Only 47% observed in latest survey [12]. Then, is it not better to classify 8 ANC visit by less than 4 and 4 and more? A woman may attend eight or more visits but still miss critical components such as blood pressure monitoring, essential tests, or comprehensive counseling on danger signs. How do you justify this? Do you consider 8 visits ensure quality ANC? Won’t it be better to consider both variables as an interactive variable to predict better service utilization for delivery?

**Authors’ response:** We thank the reviewer for the detailed comments. We would like to clarify that our study is focused on abortion and its determinants, not antenatal care (ANC) quality or frequency. Therefore, issues related to ANC visits, QANC, and skilled delivery utilization are outside the scope of our analysis. Missing data in our study were handled according to the specified eligibility criteria and complete cases were analyzed. We have clarified this in the Methods section to ensure transparency and reduce potential confusion regarding our study focus.

**Comment 4:** Line 246-266, if Chi-square test is used to test the association in bivariate tables, how is it significantly higher or lower proportion? Is this proportion test? Line 257 “The rates were significantly….” Have you calculated rates of SD and FD, or it is proportion like you have mentioned in the previous line?

**Authors’ response:** We sincerely thank the reviewer for this observation. In the bivariate analysis, we applied the Chi-square test to compare proportions between groups. When we referred to “significantly higher or lower rates,” we intended to describe these observed proportions rather than incidence rates. We have revised the manuscript to clearly state that these values represent group proportions, ensuring alignment with the statistical method used and avoiding any potential confusion.

**Comment 5:** Line 365 no association of danger signs and SBA and FD. What could be the reasons for Nigerian women with danger sign not turning up at safe delivery care facilities/manpower in this context? When they need more specialized care compared to normal women? Not discussed in detail.

**Authors’ response:** We thank the reviewer for this comment. Our study focuses on abortion, not skilled birth attendance or facility delivery.
