# Supplementary material for: Addressing abortion through individual and community-level determinants: Evidence from Southern Ethiopia
Source: PLoS One. 2026 Jun 17;21(6):e0349603. doi: 10.1371/journal.pone.0349603 (PMC13274891; doi:10.1371/journal.pone.0349603)
Supplement: Attachment — Submitted filename: I appreciate the authors Okbu.docx [file pone.0349603.s007.docx]

I appreciate the authors’ hard work and their clear commitment to improving the manuscript's quality. Most of the previously raised concerns have been adequately addressed. However, some issues remain regarding the multilevel hierarchical analysis, which still require further clarification. (As an example you look in this article about the multilevel analysis, “ *Neogi et al. BMC Pregnancy and Childbirth (2018) 18:33 DOI 10.1186/s12884-018-1660-1”)*

Before proceeding to the multilevel analysis, a separate bivariate analysis should be conducted and presented. Variables with a p-value < 0.1 in the bivariate analysis should then be included in the multilevel model, and the corresponding p-values should be clearly reported in the tables.

In the multilevel analysis, individual-level determinants should be examined first. Variables that showed statistically significant p-values should then be included in the subsequent analysis alongside community-level determinants. Another option is to omit the multilevel analysis and rely solely on multivariable analysis.

Line 64: The keywords “prevalence” and “cross-sectional study” should be removed, as they are overly generic and do not add specificity to the manuscript.
