# Supplementary material for: Addressing abortion through individual and community-level determinants: Evidence from Southern Ethiopia
Source: PLoS One. 2026 Jun 17;21(6):e0349603. doi: 10.1371/journal.pone.0349603 (PMC13274891; doi:10.1371/journal.pone.0349603)
Supplement: Attachment — Submitted filename: Response_to_Reviewers_auresp_2.docx [file pone.0349603.s009.docx]

***Point-by-Point Response to Reviewers’ and Editor Comments***

**Title:** *Addressing Abortion through Individual and Community-Level Determinants: Evidence from Southern Ethiopia*

**Manuscript ID:** *PONE-D-25-55056*

**Reviewer 1**

**Comment 1:** majority of the raised comments were addressed, but still statistical analysis was not rigorous. There was no a P value displayed in the tables. I appreciate the authors’ hard work and their clear commitment to improving the manuscript's quality. Most of the previously raised concerns have been adequately addressed. However, some issues remain regarding the multilevel hierarchical analysis, which still require further clarification. (As an example you look in this article about the multilevel analysis, “ *Neogi et al. BMC Pregnancy and Childbirth (2018) 18:33 DOI 10.1186/s12884-018-1660-1”)*

Before proceeding to the multilevel analysis, a separate bivariate analysis should be conducted and presented. Variables with a p-value < 0.1 in the bivariate analysis should then be included in the multilevel model, and the corresponding p-values should be clearly reported in the tables.

In the multilevel analysis, individual-level determinants should be examined first. Variables that showed statistically significant p-values should then be included in the subsequent analysis alongside community-level determinants. Another option is to omit the multilevel analysis and rely solely on multivariable analysis.

**Authors’ response:** We thank the reviewer for the thoughtful and constructive feedback on our statistical analysis. We have now revised the manuscript to improve clarity and rigor. We added p-values to Table 3 and 4 for both bivariate and multilevel analyses to ensure full statistical transparency. We also confirm that we conducted bivariable analysis as part of our modeling process. In the revised manuscript, we clearly present these results as adjusted odds ratios with corresponding p-values. In building the multilevel model, we included variables with p < 0.10 in bivariable analysis alongside variables supported by established evidence, following standard practice in multilevel epidemiological studies. Finally, we clarify that we retained the multilevel approach because our multistage sampling and data are hierarchically structured, and the ICC (15.71%) confirms meaningful clustering at *kebele* level. Therefore, we believe multilevel modeling is necessary and appropriate for valid inference.

**Comment 2:** Line 64: The keywords “prevalence” and “cross-sectional study” should be removed, as they are overly generic and do not add specificity to the manuscript.

**Authors’ response:** We appreciate the reviewer’s helpful suggestion. In response, we removed the keywords “prevalence” and “cross-sectional study” and revised the keyword list to make it more specific and better aligned with the focus of the manuscript.

**Reviewer 2**

**Comment 1:** Dear author, I am very sorry about my last review which was meant for some other article. You should have mentioned this in your response to the editor. The topic is important and appropriately analyzed using robust multilevel modeling approach. There some discrepancies in the manuscript as per my understanding which needs to be clarified before being accepted for publication. Spontaneous and induced abortions can have differing underlying causes. An association between, for example, "low mass media exposure" and overall "abortion" does not reveal whether media exposure prevents miscarriages or reduces the need for induced abortion. This combining of both types of abortion lead to ambiguity that might limit the validity of its conclusions. Hence the discussion and conclusion sections should be explicitly mention that observed associations cannot clearly attribute to causal pathway for either one.

**Authors’ response:** I thank the reviewer for this important clarification and fully agree with the concern. In my study, I combined spontaneous and induced abortions due to limitations in self-reported community data, where distinguishing between the two is often unreliable. However, I recognize that this may introduce ambiguity in interpreting the associations. To address this, I have revised the Discussion and Conclusion sections to clearly state that the observed associations cannot be attributed to specific causal pathways for either spontaneous or induced abortion. I also emphasize that social and community-level factors identified in this study should be interpreted cautiously, and I highlight the need for future research that separates these outcomes.

**Comment 2:** Please make clear whether the study includes only women who delivered (i.e., live births) or also women who aborted without a subsequent delivery. The eligibility criteria (lines 160, 161) and methods (page 17) states women who "had reported pregnancy that ended in abortion within same period” but the abstract (line 34, page 14) states "3,526 women of reproductive age who had delivered within the past 12 months”.

**Authors’ response:** We thank the reviewer for noting this inconsistency. We have clarified that we included all women of reproductive age who experienced any pregnancy within the past 12 months, regardless of outcome (live birth, abortion, or stillbirth). I revised the abstract and methods to ensure consistent and precise wording throughout.

**Comment 3:** It is better to provide detailed sample size calculations that account for the expected clustering effect when calculating the minimal detectable effect size for community-level variables in this design. Thank you.

**Authors’ response:** We thank the reviewer for this helpful suggestion. We have revised the Methods section to clearly describe the sample size calculation, including the design effect to account for clustering. I also clarified that the final sample size provides adequate power to detect both individual- and community-level effects in the multilevel analysis.
