# Supplementary material for: Addressing abortion through individual and community-level determinants: Evidence from Southern Ethiopia
Source: PLoS One. 2026 Jun 17;21(6):e0349603. doi: 10.1371/journal.pone.0349603 (PMC13274891; doi:10.1371/journal.pone.0349603)
Supplement: Peer review history

# Peer review: Decision Letter 0

**DOI:** 10.1371/journal.pone.0349603.r001

**Authors:** Susanne Grylka-Baeschlin

3 Mar 2026

PONE-D-25-55056

Addressing Abortion through Individual and Community-Level Determinants: Evidence from Southern Ethiopia

PLOS One

Dear Dr. Yoseph,

Thank you for submitting your manuscript to PLOS ONE. After careful consideration, we feel that it has merit but does not fully meet PLOS ONE’s publication criteria as it currently stands. Therefore, we invite you to submit a revised version of the manuscript that addresses the points raised during the review process.

Please submit your revised manuscript by Apr 17 2026 11:59PM. If you will need more time than this to complete your revisions, please reply to this message or contact the journal office at plosone@plos.org. When you're ready to submit your revision, log on to https://www.editorialmanager.com/pone/ and select the 'Submissions Needing Revision' folder to locate your manuscript file.

Please include the following items when submitting your revised manuscript:

- A letter that responds to each point raised by the academic editor and reviewer(s). You should upload this letter as a separate file labeled 'Response to Reviewers'.
- A marked-up copy of your manuscript that highlights changes made to the original version. You should upload this as a separate file labeled 'Revised Manuscript with Track Changes'.
- An unmarked version of your revised paper without tracked changes. You should upload this as a separate file labeled 'Manuscript'.

If you would like to make changes to your financial disclosure, please include your updated statement in your cover letter. Guidelines for resubmitting your figure files are available below the reviewer comments at the end of this letter.

If applicable, we recommend that you deposit your laboratory protocols in protocols.io to enhance the reproducibility of your results. Protocols.io assigns your protocol its own identifier (DOI) so that it can be cited independently in the future. For instructions see: https://journals.plos.org/plosone/s/submission-guidelines#loc-laboratory-protocols. Additionally, PLOS ONE offers an option for publishing peer-reviewed Lab Protocol articles, which describe protocols hosted on protocols.io. Read more information on sharing protocols at https://plos.org/protocols?utm_medium=editorial-email&utm_source=authorletters&utm_campaign=protocols.

We look forward to receiving your revised manuscript.

Kind regards,

Susanne Grylka-Baeschlin, PhD

Academic Editor

PLOS One

Journal Requirements:

When submitting your revision, we need you to address these additional requirements.

1. Please ensure that your manuscript meets PLOS ONE's style requirements, including those for file naming. The PLOS ONE style templates can be found at

https://journals.plos.org/plosone/s/file?id=wjVg/PLOSOne_formatting_sample_main_body.pdf and

https://journals.plos.org/plosone/s/file?id=ba62/PLOSOne_formatting_sample_title_authors_affiliations.pdf

2. Thank you for stating the following in the Acknowledgments Section of your manuscript:

“This study was supported by Nestlé Foundation with grant number of HUHS/021/2016. The funders had no role in the design and conduct of the study; collection, management, analysis, and interpretation of the data; preparation, review, or approval of the manuscript; and decision to submit the manuscript for publication.”

We note that you have provided funding information that is not currently declared in your Funding Statement. However, funding information should not appear in the Acknowledgments section or other areas of your manuscript. We will only publish funding information present in the Funding Statement section of the online submission form.

Please remove any funding-related text from the manuscript and let us know how you would like to update your Funding Statement. Currently, your Funding Statement reads as follows:

“This study was supported by Nestlé Foundation with grant number of HUHS/021/2016. The funders had no role in the design and conduct of the study; collection, management, analysis, and interpretation of the data; preparation, review, or approval of the manuscript; and decision to submit the manuscript for publication.”

Please include your amended statements within your cover letter; we will change the online submission form on your behalf.

3. Please include a separate caption for each figure in your manuscript.

4. Thank you for providing your underlying data as Supporting Information.

We note that the data set contains text or data that is not in English. Please note that PLOS is an English-language publisher, so we require data sets to be provided in English as well. Please upload an English-language version of your data set.

This will also allow us to determine if your data follows PLOS standards per our Data Availability policy here: https://journals.plos.org/plosone/s/data-availability

5. If the reviewer comments include a recommendation to cite specific previously published works, please review and evaluate these publications to determine whether they are relevant and should be cited. There is no requirement to cite these works unless the editor has indicated otherwise.

[Note: HTML markup is below. Please do not edit.]

Reviewers' comments:

Reviewer's Responses to Questions

**Comments to the Author**

1. Is the manuscript technically sound, and do the data support the conclusions?

The manuscript must describe a technically sound piece of scientific research with data that supports the conclusions. Experiments must have been conducted rigorously, with appropriate controls, replication, and sample sizes. The conclusions must be drawn appropriately based on the data presented.

Reviewer #1: Partly

Reviewer #2: Partly

**********

2. Has the statistical analysis been performed appropriately and rigorously?

Reviewer #1: No

Reviewer #2: No

**********

3. Have the authors made all data underlying the findings in their manuscript fully available?

The PLOS Data policy requires authors to make all data underlying the findings described in their manuscript fully available without restriction, with rare exception (please refer to the Data Availability Statement in the manuscript PDF file). The data should be provided as part of the manuscript or its supporting information, or deposited to a public repository. For example, in addition to summary statistics, the data points behind means, medians and variance measures should be available. If there are restrictions on publicly sharing data—e.g. participant privacy or use of data from a third party—those must be specified.

Reviewer #1: Yes

Reviewer #2: No

**********

4. Is the manuscript presented in an intelligible fashion and written in standard English?

PLOS ONE does not copyedit accepted manuscripts, so the language in submitted articles must be clear, correct, and unambiguous. Any typographical or grammatical errors should be corrected at revision, so please note any specific errors here.

Reviewer #1: Yes

Reviewer #2: Yes

**********

5. Review Comments to the Author

Please use the space provided to explain your answers to the questions above. You may also include additional comments for the author, including concerns about dual publication, research ethics, or publication ethics. (Please upload your review as an attachment if it exceeds 20,000 characters)

Reviewer #1: The overall idea of multimodal analysis is awe-inspiring, but not fully explored. And the study would be attractive if it were concentrated on a specific abortion (induced abortion). At least 15% of pregnancies ended up in abortion, the majority caused by chromosomal abnormality, where the community level has no role in determining their occurrence.

Methodology

In line 179. Parity refers to the number of live births, which is not necessarily the number of live births. Even a stillbirth was a parity.

Statistical analysis

About the pregnancy status, it would be better to use the planned delivery as a reference.

About the Woman's decision-making power, it would be better to use Autonomous as a reference.

In community-level distance to nearest health facility, it would be better to use not a big problem as a reference.

Variables with p < 0.25 in bivariable analysis or established clinical relevance were included in the multivariable model. The P <0.25 could increase the confounders. I would suggest taking P <0.1 at bivariate analysis to put them in a hierarchical multimodal approach.

Operational definition should be included to address the following:-

Community-level mass media use

Wealth quintile needs operational definitions

Obstetric danger signs during pregnancy

Sampling procedure

The sampling procedure would be informative if presented in a plotted figure.

Eligibility Criteria

Eligibility Criteria: if a resident stays for at least six months and reports a pregnancy that ended in abortion within the past year, this criterion does not necessarily require the abortion to occur within the district. To explain the community level determinants, it should reside for at least one year.

Ethical Considerations

The reference number should be included.

Results

1. The results stated that at the community level, rural residence and high community-level mass media exposure was linked with higher odds of abortion was associated with increased odds of abortion. The high community-level mass media exposure was probably in urban areas, and this contradiction needs to be clearly addressed.

2. A table should be prepared for multilevel logistic regression analysis.

Reviewer #2: Dear Author,

This study addresses gap by examining a qualitative and quantity of ANC in skilled delivery utilization care thereby contributing to the maternal health literature in Bangladesh and similar LMIC settings. and appropriate multilevel modeling techniques. There are minor queries and issues regarding the definition of outcome and study variable's definition and how does this impact to the primary findings of the study. Please see the attached document for full detail.

Regards

**********

6. PLOS authors have the option to publish the peer review history of their article (what does this mean?). If published, this will include your full peer review and any attached files.

If you choose “no”, your identity will remain anonymous but your review may still be made public.

**Do you want your identity to be public for this peer review?**  For information about this choice, including consent withdrawal, please see our Privacy Policy.

Reviewer #1: **Yes:** Okbu Frezgi, MD. Obstetrician and Gynecologist.

Orotta College of Medicine and Health Sciences

Reviewer #2: **Yes:** Dr ailesh Bhattarai Ph.D. Public Health

**********

[NOTE: If reviewer comments were submitted as an attachment file, they will be attached to this email and accessible via the submission site. Please log into your account, locate the manuscript record, and check for the action link "View Attachments". If this link does not appear, there are no attachment files.]

To ensure your figures meet our technical requirements, please review our figure guidelines: https://journals.plos.org/plosone/s/figures

You may also use PLOS’s free figure tool, NAAS, to help you prepare publication quality figures: https://journals.plos.org/plosone/s/figures#loc-tools-for-figure-preparation.

NAAS will assess whether your figures meet our technical requirements by comparing each figure against our figure specifications.

Attachment Submitted filename: Pone-D-25-03111_reviewer.docx

# Author comment: Author response to Decision Letter 1

**DOI:** 10.1371/journal.pone.0349603.r002

16 Mar 2026

Point-by-Point Response to Reviewers’ and Editor Comments

Title: Addressing Abortion through Individual and Community-Level Determinants: Evidence from Southern Ethiopia

Manuscript ID: PONE-D-25-55056

Journal Requirements

Comment 1. Please ensure that your manuscript meets PLOS ONE's style requirements, including those for file naming. The PLOS ONE style templates can be found at https://journals.plos.org/plosone/s/file?id=wjVg/PLOSOne_formatting_sample_main_body.pdf and https://journals.plos.org/plosone/s/file?id=ba62/PLOSOne_formatting_sample_title_authors_affiliations.pdf

Authors’ response: Thank you for this comment. We have reviewed the manuscript and revised it to ensure full compliance with the PLOS ONE formatting and style requirements, including file naming, manuscript structure, and the presentation of the title page and main text according to the journal’s templates.

Comment 2. Thank you for stating the following in the Acknowledgments Section of your manuscript: “This study was supported by Nestlé Foundation with grant number of HUHS/021/2016. The funders had no role in the design and conduct of the study; collection, management, analysis, and interpretation of the data; preparation, review, or approval of the manuscript; and decision to submit the manuscript for publication.”

We note that you have provided funding information that is not currently declared in your Funding Statement. However, funding information should not appear in the Acknowledgments section or other areas of your manuscript. We will only publish funding information present in the Funding Statement section of the online submission form.

Please remove any funding-related text from the manuscript and let us know how you would like to update your Funding Statement. Currently, your Funding Statement reads as follows: “This study was supported by Nestlé Foundation with grant number of HUHS/021/2016. The funders had no role in the design and conduct of the study; collection, management, analysis, and interpretation of the data; preparation, review, or approval of the manuscript; and decision to submit the manuscript for publication.” Please include your amended statements within your cover letter; we will change the online submission form on your behalf.

Authors’ response: Thank you for this clarification. In line with the journal’s guidance, we have removed the funding information from the Acknowledgments section. The correct funding source is the Sidama Region President Office (Grant No. SPO/123/16), and this information should appear only in the Funding Statement of the submission system. The funder had no role in the study design, data collection, analysis, interpretation, manuscript preparation, or the decision to submit the manuscript.

Comment 3. Please include a separate caption for each figure in your manuscript.

Authors’ response: Thank you for this comment. We have revised the manuscript to ensure that each figure now includes a separate caption, in accordance with PLOS ONE formatting requirements.

Comment 4. Thank you for providing your underlying data as Supporting Information. We note that the data set contains text or data that is not in English. Please note that PLOS is an English-language publisher, so we require data sets to be provided in English as well. Please upload an English-language version of your data set. This will also allow us to determine if your data follows PLOS standards per our Data Availability policy here: https://journals.plos.org/plosone/s/data-availability

Authors’ response: Thank you for this comment. We have carefully translated the dataset into English and uploaded the revised English-language version as Supporting Information to fully comply with PLOS ONE’s Data Availability policy.

Comment 5. If the reviewer comments include a recommendation to cite specific previously published works, please review and evaluate these publications to determine whether they are relevant and should be cited. There is no requirement to cite these works unless the editor has indicated otherwise.

Authors’ response: Thank you for the guidance. The reviewers did not recommend any specific references, and after careful consideration, we did not add any additional citations to the manuscript.

Reviewer 1

Comment 1: The overall idea of multimodal analysis is awe-inspiring but not fully explored. And the study would be attractive if it were concentrated on a specific abortion (induced abortion). At least 15% of pregnancies ended up in abortion, the majority caused by chromosomal abnormality, where the community level has no role in determining their occurrence.

Authors’ response: Thank you for this important comment. We acknowledge that abortions can result from diverse causes, including biological factors such as chromosomal abnormalities, which primarily account for early spontaneous abortions and are not influenced by community-level factors. However, our study examines abortions as reported by women in the community, encompassing both induced and spontaneous cases, to understand the broader social and environmental determinants of reproductive outcomes.

In settings like Hawela Lida District, a substantial proportion of abortions are likely influenced by preventable or socially mediated factors, including unintended pregnancies, limited autonomy in decision-making, restricted access to healthcare, and sociocultural pressures. Community-level factors such as rural residence, proximity to health facilities, and exposure to mass media - can shape reproductive behaviors and access to family planning, thereby affecting the occurrence of abortions that are socially or behaviorally mediated.

While biological causes contribute to some spontaneous abortions, our findings show that many cases are influenced by factors that can be addressed through public health interventions. We have revised the manuscript to clearly acknowledge the limits of community-level influence on biologically determined abortions while highlighting the relevance of multilevel determinants for most cases observed in this population.

Methodology

Comment 2: In line 179. Parity refers to the number of live births, which is not necessarily the number of live births. Even a stillbirth was a parity.

Authors’ response: Thank you for highlighting this point. We acknowledge that, by definition, parity refers to the number of pregnancies reaching a viable gestational age, including live births, stillbirths, and sometimes multiple births. In our study, we carefully measured parity as the total number of pregnancies that progressed to ≥28 weeks gestation, thereby including both live births and stillbirths. We have clarified this definition in the Methods section to accurately reflect standard obstetric terminology and ensure consistency with established reporting practices.

Statistical analysis

Comment 3: About the pregnancy status, it would be better to use the planned delivery as a reference.

Authors’ response: Thank you for this suggestion. We agree that using planned pregnancies as the reference category provides clearer interpretability of the odds ratios and aligns with common epidemiological practice. Accordingly, we have updated our multivariable analysis so that planned pregnancies now serve as the reference group, and the results have been revised in Table 3 and throughout the text to reflect this change.

Comment 4: About the Woman's decision-making power, it would be better to use Autonomous as a reference.

Authors’ response: Thank you for this recommendation. We agree that using autonomous women as the reference category provides clearer interpretability and aligns with standard analytical practice. Accordingly, we have updated our multivariable analysis so that autonomous women now serve as the reference group. All relevant results in Table 3 and throughout the manuscript have been revised to reflect this change.

Comment 5: In community-level distance to nearest health facility, it would be better to use not a big problem as a reference.

Authors’ response: Thank you for this suggestion. We agree that using “not a big problem” as the reference category for community-level distance to the nearest health facility enhances interpretability of the results. We have updated our multivariable analysis accordingly, and all relevant results in Table 3 and the text have been revised to reflect this change.

Comment 6: Variables with p < 0.25 in bivariable analysis or established clinical relevance were included in the multivariable model. The P <0.25 could increase the confounders. I would suggest taking P <0.1 at bivariate analysis to put them in a hierarchical multimodal approach.

Authors’ response: Thank you for your valuable suggestion. We have updated the Statistical Analysis section to reflect your recommendation, clarifying that variables with p < 0.10 at the bivariable stage or with established clinical relevance were included in the multivariable model. Upon review, we found that all variables in our analysis already met this threshold, ensuring consistency with a hierarchical multimodal approach.

Comment 7: Operational definition should be included to address the following: Community-level mass media use, wealth quintile needs operational definitions and obstetric danger signs during pregnancy

Authors’ response: Thank you for the comment. We have added operational definitions for the requested variables: community-level mass media use, wealth quintile, and obstetric danger signs. These are now included in the Methods section.

Sampling procedure

Comment 8: The sampling procedure would be informative if presented in a plotted figure.

Authors’ response: Thank you for this suggestion. We have now created a flowchart illustrating the multistage sampling procedure, from kebele selection to household and participant inclusion. This figure has been added as Figure 2 in the revised manuscript to improve clarity and reader comprehension.

Eligibility Criteria

Comment 9: Eligibility Criteria: if a resident stays for at least six months and reports a pregnancy that ended in abortion within the past year, this criterion does not necessarily require the abortion to occur within the district. To explain the community level determinants, it should reside for at least one year.

Authors’ response: We appreciate this insightful comment. In response, we have updated the eligibility criteria so that women must have resided in Hawela Lida District for at least one year and report a pregnancy that ended in abortion within that period. This modification ensures that all participants have sufficient exposure to the community context, thereby strengthening the validity of our assessment of community-level determinants.

Ethical Considerations

Comment 10: The reference number should be included.

Authors’ response: Thank you for this comment. We have now included the ethical approval reference number in the manuscript. Ethical approval was obtained from the Institutional Review Board of the College of Medicine and Health Sciences, Hawassa University (Ref: /IRB/027/17).

Results

Comment 11. The results stated that at the community level, rural residence and high community-level mass media exposure was linked with higher odds of abortion was associated with increased odds of abortion. The high community-level mass media exposure was probably in urban areas, and this contradiction needs to be clearly addressed.

Authors’ response: We appreciate the reviewer’s careful observation. Upon review, we realized that the reference was mistakenly assigned in the original manuscript. This has now been corrected to accurately reflect the appropriate source, ensuring clarity and consistency in the text.

Comment 12. A table should be prepared for multilevel logistic regression analysis.

Authors’ response: We sincerely thank the reviewer for this constructive suggestion. In response, we have created a comprehensive table presenting the results of the multilevel logistic regression analysis, incorporating both individual- and community-level determinants, along with the corresponding adjusted odds ratios and 95% confidence intervals. This table has been added to the revised manuscript to enhance clarity and facilitate interpretation of our findings.

Reviewer 2

General comment: This study addresses gap by examining a qualitative and quantity of ANC in skilled delivery utilization care thereby contributing to the maternal health literature in Bangladesh and similar LMIC settings. and appropriate multilevel modeling techniques. There are minor queries and issues regarding the definition of outcome and study variable's definition and how does this impact to the primary findings of the study. Please see the attached document for full detail.

Authors’ response: We thank the reviewer for acknowledging the significance of our study and the appropriateness of the multilevel modeling approach. We have carefully reviewed all the minor queries regarding outcome definitions and variable operationalization and have addressed them in the revised manuscript. These clarifications do not alter the primary findings but strengthen the transparency and interpretability of our results.

Comment 1: The study rationales receiving high–quality ANC interacts with the updated WHO recommendations to improve the use of SBA and facility deliveries”. This study has not been explicitly reported in the study findings. If I have missed please explain where the quality and quantity of ANC services combine to give interactive effect on health care delivery service utilization.

Authors’ response: We thank the reviewer for this comment. Our study focuses on abortion and its determinants; issues related to ANC quality or facility deliveries are beyond its scope. The manuscript has been revised to clarify this focus.

Comment 2: How was 675 EAs chosen? The basis for choosing exactly that number. The second stage was EA cluster. Why was not design effect not applied? “variables coded as 1 (good QANC) if the pregnant women received all seven essential ANC components at least once and 2 (poor QANC) otherwise” What is the justification of this stringent criteria of all/none (can it not be in some cases one of the components were not fulfilled because it was not indicated?) how do you justify it.? The good QANC is only 21.6% probably need to be rechecked using sensitivity analysis using (0-7) score or different cutoffs to test the robustness. ANC visit categorization is (1-3) overlaps between inadequate and almost adequate (WHO recommended 4). Justify if three visits are considered in adequate. What percentage of SBA codded 1 had FD code 1? This looks like overlapping variables.

Authors’ response: We appreciate the reviewer’s detailed feedback. However, our study focuses on abortion and its determinants, not ANC quality or skilled birth attendance. The points raised regarding ANC components, EA selection, design effect, and variable coding pertain to maternal care studies, which are outside the scope of our current analysis. We have clarified this focus throughout the manuscript to avoid confusion.

Comment 3: How is missing data handled? (complete case analysis? number excluded? etc). Mention about potential bias occurring out of from excluding flooded clusters. WHO recommends 8 visits just to ensure quality antenatal checkups. If ultimately, we have to rely on QANC as valid indicator to ensure proper delivery service utilization then why 8 ANC is taken as a second key exposure when QNC means better indicator whether 4 six or 8 Visits are done. Moreover, Bangladesh’s national guidelines still promote four or more ANC visits [13], then we know low quality service utilization is because of national policy. Only 47% observed in latest survey [12]. Then, is it not better to classify 8 ANC visit by less than 4 and 4 and more? A woman may attend eight or more visits but still miss critical components such as blood pressure monitoring, essential tests, or comprehensive counseling on danger signs. How do you justify this? Do you consider 8 visits ensure quality ANC? Won’t it be better to consider both variables as an interactive variable to predict better service utilization for delivery

Attachment Submitted filename: Response to Reviewers.docx

# Peer review: Decision Letter 1

**DOI:** 10.1371/journal.pone.0349603.r003

**Authors:** Susanne Grylka-Baeschlin

11 Apr 2026

<div>PONE-D-25-55056R1

Addressing Abortion through Individual and Community-Level Determinants: Evidence from Southern Ethiopia

PLOS One

Dear Dr. Yoseph,

Thank you for submitting your manuscript to PLOS ONE. After careful consideration, we feel that it has merit but does not fully meet PLOS ONE’s publication criteria as it currently stands. Therefore, we invite you to submit a revised version of the manuscript that addresses the points raised during the review process.

Please submit your revised manuscript by May 26 2026 11:59PM. If you will need more time than this to complete your revisions, please reply to this message or contact the journal office at plosone@plos.org. When you're ready to submit your revision, log on to https://www.editorialmanager.com/pone/ and select the 'Submissions Needing Revision' folder to locate your manuscript file.

Please include the following items when submitting your revised manuscript:

- A letter that responds to each point raised by the academic editor and reviewer(s). You should upload this letter as a separate file labeled 'Response to Reviewers'.
- A marked-up copy of your manuscript that highlights changes made to the original version. You should upload this as a separate file labeled 'Revised Manuscript with Track Changes'.
- An unmarked version of your revised paper without tracked changes. You should upload this as a separate file labeled 'Manuscript'.

If you would like to make changes to your financial disclosure, please include your updated statement in your cover letter. Guidelines for resubmitting your figure files are available below the reviewer comments at the end of this letter.

If applicable, we recommend that you deposit your laboratory protocols in protocols.io to enhance the reproducibility of your results. Protocols.io assigns your protocol its own identifier (DOI) so that it can be cited independently in the future. For instructions see: https://journals.plos.org/plosone/s/submission-guidelines#loc-laboratory-protocols. Additionally, PLOS ONE offers an option for publishing peer-reviewed Lab Protocol articles, which describe protocols hosted on protocols.io. Read more information on sharing protocols at https://plos.org/protocols?utm_medium=editorial-email&utm_source=authorletters&utm_campaign=protocols.

As the corresponding author, your ORCID iD is verified in the submission system and will appear in the published article. PLOS supports the use of ORCID, and we encourage all coauthors to register for an ORCID iD and use it as well. Please encourage your coauthors to verify their ORCID iD within the submission system before final acceptance, as unverified ORCID iDs will not appear in the published article. *Only*  the individual author can complete the verification step; PLOS staff *cannot*  verify ORCID iDs on behalf of authors.

We look forward to receiving your revised manuscript.

Kind regards,

Susanne Grylka-Baeschlin, PhD

Academic Editor

PLOS One

Journal Requirements:

If the reviewer comments include a recommendation to cite specific previously published works, please review and evaluate these publications to determine whether they are relevant and should be cited. There is no requirement to cite these works unless the editor has indicated otherwise.

Please review your reference list to ensure that it is complete and correct. If you have cited papers that have been retracted, please include the rationale for doing so in the manuscript text, or remove these references and replace them with relevant current references. Any changes to the reference list should be mentioned in the rebuttal letter that accompanies your revised manuscript. If you need to cite a retracted article, indicate the article’s retracted status in the References list and also include a citation and full reference for the retraction notice.

[Note: HTML markup is below. Please do not edit.]

Reviewers' comments:

Reviewer's Responses to Questions

**Comments to the Author**

1. If the authors have adequately addressed your comments raised in a previous round of review and you feel that this manuscript is now acceptable for publication, you may indicate that here to bypass the “Comments to the Author” section, enter your conflict of interest statement in the “Confidential to Editor” section, and submit your "Accept" recommendation.

Reviewer #1: All comments have been addressed

Reviewer #2: (No Response)

**********

2. Is the manuscript technically sound, and do the data support the conclusions?

The manuscript must describe a technically sound piece of scientific research with data that supports the conclusions. Experiments must have been conducted rigorously, with appropriate controls, replication, and sample sizes. The conclusions must be drawn appropriately based on the data presented.

Reviewer #1: Partly

Reviewer #2: Partly

**********

3. Has the statistical analysis been performed appropriately and rigorously?

Reviewer #1: No

Reviewer #2: Yes

**********

4. Have the authors made all data underlying the findings in their manuscript fully available?

The PLOS Data policy requires authors to make all data underlying the findings described in their manuscript fully available without restriction, with rare exception (please refer to the Data Availability Statement in the manuscript PDF file). The data should be provided as part of the manuscript or its supporting information, or deposited to a public repository. For example, in addition to summary statistics, the data points behind means, medians and variance measures should be available. If there are restrictions on publicly sharing data—e.g. participant privacy or use of data from a third party—those must be specified.

Reviewer #1: Yes

Reviewer #2: No

**********

5. Is the manuscript presented in an intelligible fashion and written in standard English?

PLOS ONE does not copyedit accepted manuscripts, so the language in submitted articles must be clear, correct, and unambiguous. Any typographical or grammatical errors should be corrected at revision, so please note any specific errors here.

Reviewer #1: Yes

Reviewer #2: Yes

**********

6. Review Comments to the Author

Please use the space provided to explain your answers to the questions above. You may also include additional comments for the author, including concerns about dual publication, research ethics, or publication ethics. (Please upload your review as an attachment if it exceeds 20,000 characters)

Reviewer #1: majority of the raised comments were addressed, but still statistical analysis was not rigorous. There was no a P value displayed in the tables.

Reviewer #2: Dear author,

I am very sorry about my last review which was meant for some other article. You should have mentioned this in your response to the editor. The topic is important and appropriately analyzed using robust multilevel modeling approach. There some discrepancies in the manuscript as per my understanding which needs to be clarified before being accepted for publication.

Spontaneous and induced abortions can have differing underlying causes. An association between, for example, "low mass media exposure" and overall "abortion" does not reveal whether media exposure prevents miscarriages or reduces the need for induced abortion. This combining of both types of abortion lead to ambiguity that might limit the validity of its conclusions. Hence the discussion and conclusion sections should be explicitly mention that observed associations cannot clearly attribute to causal pathway for either one.

Please make clear whether the study includes only women who delivered (i.e., live births) or also women who aborted without a subsequent delivery. The eligibility criteria (lines 160, 161) and methods (page 17) states women who "had reported pregnancy that ended in abortion within same period” but the abstract (line 34, page 14) states "3,526 women of reproductive age who had delivered within the past 12 months”.

It is better to provide detailed sample size calculations that account for the expected clustering effect when calculating the minimal detectable effect size for community-level variables in this design.

Thank you.

**********

7. PLOS authors have the option to publish the peer review history of their article (what does this mean?). If published, this will include your full peer review and any attached files.

If you choose “no”, your identity will remain anonymous but your review may still be made public.

**Do you want your identity to be public for this peer review?**  For information about this choice, including consent withdrawal, please see our Privacy Policy.

Reviewer #1: **Yes:** Dr. Okbu Frezgi

Reviewer #2: **Yes:** Dr. Sailesh Bhattarai Ph.D. Public Health

**********

[NOTE: If reviewer comments were submitted as an attachment file, they will be attached to this email and accessible via the submission site. Please log into your account, locate the manuscript record, and check for the action link "View Attachments". If this link does not appear, there are no attachment files.]

To ensure your figures meet our technical requirements, please review our figure guidelines: https://journals.plos.org/plosone/s/figures

You may also use PLOS’s free figure tool, NAAS, to help you prepare publication quality figures: https://journals.plos.org/plosone/s/figures#loc-tools-for-figure-preparation.

NAAS will assess whether your figures meet our technical requirements by comparing each figure against our figure specifications.

Attachment Submitted filename: I appreciate the authors Okbu.docx

# Author comment: Author response to Decision Letter 2

**DOI:** 10.1371/journal.pone.0349603.r004

15 Apr 2026

Point-by-Point Response to Reviewers’ and Editor Comments

Title: Addressing Abortion through Individual and Community-Level Determinants: Evidence from Southern Ethiopia

Manuscript ID: PONE-D-25-55056

Reviewer 1

Comment 1: majority of the raised comments were addressed, but still statistical analysis was not rigorous. There was no a P value displayed in the tables. I appreciate the authors’ hard work and their clear commitment to improving the manuscript's quality. Most of the previously raised concerns have been adequately addressed. However, some issues remain regarding the multilevel hierarchical analysis, which still require further clarification. (As an example you look in this article about the multilevel analysis, “ Neogi et al. BMC Pregnancy and Childbirth (2018) 18:33 DOI 10.1186/s12884-018-1660-1”)

Before proceeding to the multilevel analysis, a separate bivariate analysis should be conducted and presented. Variables with a p-value < 0.1 in the bivariate analysis should then be included in the multilevel model, and the corresponding p-values should be clearly reported in the tables.

In the multilevel analysis, individual-level determinants should be examined first. Variables that showed statistically significant p-values should then be included in the subsequent analysis alongside community-level determinants. Another option is to omit the multilevel analysis and rely solely on multivariable analysis.

Authors’ response: We thank the reviewer for the thoughtful and constructive feedback on our statistical analysis. We have now revised the manuscript to improve clarity and rigor. We added p-values to Table 3 and 4 for both bivariate and multilevel analyses to ensure full statistical transparency. We also confirm that we conducted bivariable analysis as part of our modeling process. In the revised manuscript, we clearly present these results as adjusted odds ratios with corresponding p-values. In building the multilevel model, we included variables with p < 0.10 in bivariable analysis alongside variables supported by established evidence, following standard practice in multilevel epidemiological studies. Finally, we clarify that we retained the multilevel approach because our multistage sampling and data are hierarchically structured, and the ICC (15.71%) confirms meaningful clustering at kebele level. Therefore, we believe multilevel modeling is necessary and appropriate for valid inference.

Comment 2: Line 64: The keywords “prevalence” and “cross-sectional study” should be removed, as they are overly generic and do not add specificity to the manuscript.

Authors’ response: We appreciate the reviewer’s helpful suggestion. In response, we removed the keywords “prevalence” and “cross-sectional study” and revised the keyword list to make it more specific and better aligned with the focus of the manuscript.

Reviewer 2

Comment 1: Dear author, I am very sorry about my last review which was meant for some other article. You should have mentioned this in your response to the editor. The topic is important and appropriately analyzed using robust multilevel modeling approach. There some discrepancies in the manuscript as per my understanding which needs to be clarified before being accepted for publication. Spontaneous and induced abortions can have differing underlying causes. An association between, for example, "low mass media exposure" and overall "abortion" does not reveal whether media exposure prevents miscarriages or reduces the need for induced abortion. This combining of both types of abortion lead to ambiguity that might limit the validity of its conclusions. Hence the discussion and conclusion sections should be explicitly mention that observed associations cannot clearly attribute to causal pathway for either one.

Authors’ response: I thank the reviewer for this important clarification and fully agree with the concern. In my study, I combined spontaneous and induced abortions due to limitations in self-reported community data, where distinguishing between the two is often unreliable. However, I recognize that this may introduce ambiguity in interpreting the associations. To address this, I have revised the Discussion and Conclusion sections to clearly state that the observed associations cannot be attributed to specific causal pathways for either spontaneous or induced abortion. I also emphasize that social and community-level factors identified in this study should be interpreted cautiously, and I highlight the need for future research that separates these outcomes.

Comment 2: Please make clear whether the study includes only women who delivered (i.e., live births) or also women who aborted without a subsequent delivery. The eligibility criteria (lines 160, 161) and methods (page 17) states women who "had reported pregnancy that ended in abortion within same period” but the abstract (line 34, page 14) states "3,526 women of reproductive age who had delivered within the past 12 months”.

Authors’ response: We thank the reviewer for noting this inconsistency. We have clarified that we included all women of reproductive age who experienced any pregnancy within the past 12 months, regardless of outcome (live birth, abortion, or stillbirth). I revised the abstract and methods to ensure consistent and precise wording throughout.

Comment 3: It is better to provide detailed sample size calculations that account for the expected clustering effect when calculating the minimal detectable effect size for community-level variables in this design. Thank you.

Authors’ response: We thank the reviewer for this helpful suggestion. We have revised the Methods section to clearly describe the sample size calculation, including the design effect to account for clustering. I also clarified that the final sample size provides adequate power to detect both individual- and community-level effects in the multilevel analysis.

Attachment Submitted filename: Response_to_Reviewers_auresp_2.docx

# Editor report: Decision Letter 2

**DOI:** 10.1371/journal.pone.0349603.r005

**Authors:** Susanne Grylka-Baeschlin

4 May 2026

<p>Addressing Abortion through Individual and Community-Level Determinants: Evidence from Southern Ethiopia

PONE-D-25-55056R2

Dear Dr. Yoseph,

We’re pleased to inform you that your manuscript has been judged scientifically suitable for publication and will be formally accepted for publication once it meets all outstanding technical requirements.

Within one week, you’ll receive an e-mail detailing the required amendments. When these have been addressed, you’ll receive a formal acceptance letter and your manuscript will be scheduled for publication.

An invoice will be generated when your article is formally accepted. Please note, if your institution has a publishing partnership with PLOS and your article meets the relevant criteria, all or part of your publication costs will be covered. Please make sure your user information is up-to-date by logging into Editorial Manager at Editorial Manager® and clicking the ‘Update My Information' link at the top of the page. For questions related to billing, please contact billing support.

If your institution or institutions have a press office, please notify them about your upcoming paper to help maximize its impact. If they’ll be preparing press materials, please inform our press team as soon as possible -- no later than 48 hours after receiving the formal acceptance. Your manuscript will remain under strict press embargo until 2 pm Eastern Time on the date of publication. For more information, please contact onepress@plos.org.

Kind regards,

Susanne Grylka-Baeschlin, PhD

Academic Editor

PLOS One

Additional Editor Comments (optional):

– Conclusion (abstract): “Abortion in Hawela Lida District was associated …” instead of “is associated”.

– Methods, page 5, line 134: “ensured” instead of “ensures”.

– Conclusion (manuscript), page 23, line 439: again “was associated”.

Reviewers' comments:

# Editor report: Acceptance letter

**DOI:** 10.1371/journal.pone.0349603.r006

**Authors:** Susanne Grylka-Baeschlin

PONE-D-25-55056R2

PLOS One

Dear Dr. Yoseph,

I'm pleased to inform you that your manuscript has been deemed suitable for publication in PLOS One. Congratulations! Your manuscript is now being handed over to our production team.

At this stage, our production department will prepare your paper for publication. This includes ensuring the following:

* All references, tables, and figures are properly cited

* All relevant supporting information is included in the manuscript submission,

* There are no issues that prevent the paper from being properly typeset

You will receive further instructions from the production team, including instructions on how to review your proof when it is ready. Please keep in mind that we are working through a large volume of accepted articles, so please give us a few days to review your paper and let you know the next and final steps.

Lastly, if your institution or institutions have a press office, please let them know about your upcoming paper now to help maximize its impact. If they'll be preparing press materials, please inform our press team within the next 48 hours. Your manuscript will remain under strict press embargo until 2 pm Eastern Time on the date of publication. For more information, please contact onepress@plos.org.

You will receive an invoice from PLOS for your publication fee after your manuscript has reached the completed accept phase. If you receive an email requesting payment before acceptance or for any other service, this may be a phishing scheme. Learn how to identify phishing emails and protect your accounts at https://explore.plos.org/phishing.

If we can help with anything else, please email us at customercare@plos.org.

Thank you for submitting your work to PLOS ONE and supporting open access.

Kind regards,

PLOS ONE Editorial Office Staff

on behalf of

Prof. Dr. Susanne Grylka-Baeschlin

Academic Editor

PLOS One
